# Thermal cycles behavior and microstructure of AZ31/SiC composite prepared by stir casting

**DOI:** 10.1038/s41598-022-19410-2

**Published:** 2022-09-07

**Authors:** Seyed Fereidon Mousavi, Hassan Sharifi, Morteza Tayebi, Bejan Hamawandi, Yashar Behnamian

**Affiliations:** 1 Department of Materials Science, Faculty of Engineering, University of Shahrekord, Shahrekord, Iran; 2 Young Researchers and Elites Club, Science and Research Branch, Islamic Azad University, Tehran, Iran; 3 Department of Applied Physics, KTH Royal Institute of Technology, 106 91 Stockholm, Sweden; 4 Department of Chemical and Materials Engineering, University of Alberta, Alberta, Edmonton T6G 2V4 Canada

**Keywords:** Materials for devices, Structural materials

## Abstract

In the present work, the effect of thermal cycles on the physical and thermal properties of AZ31 alloy and AZ31/5wt%SiC and AZ31/10wt%SiC composites was investigated. Samples were prepared using the stir casting method and then subjected to precipitation hardening. Thermal cycles were done for as-cast and aged samples with V-shaped notch under 300, 600, and 900 heating and cooling cycles at 150 and 350 °C. The crack length (CL) was evaluated using optical microscope (OM), scanning electron microscope (SEM), and energy-dispersive scanning electron (EDS) analysis. Also, density, porosity, thermal expansion coefficient of the samples were evaluated. X-ray diffraction (XRD) analysis was employed to assess the phases present in the material. The results demonstrated that by increasing the number of thermal cycles up to 600 at 150 °C and 350 °C, the porosity and density of the as-cast and aged AZ31 alloy decreased and increased, respectively; however, the density and open porosity were remained constant for the composite samples. The crack's length enlarged with increasing the thermal cycles from 300 to 600 µm at 150 °C and 300 to 900 µm at 350 °C. It was found that the reinforcement and precipitates prevented the rapid growth of the crack in the magnesium matrix. All in All, composite and the aged samples demonstrated better thermal fatigue resistance compared with that of the unreinforced alloy and as-cast samples, respectively.

## Introduction

Over the recent years, magnesium alloys have received a great deal of attention in aerospace, aircraft, and automotive industries on account of lightweight^1–4^. Considering lower density than other alloys, namely about two-thirds that of aluminum, one-fourth that of zinc, and one-fifth that of steel, among conventional engineering alloys, magnesium alloys offer very high strength. Furthermore, magnesium alloys possess outstanding damping capacity, excellent casting, and machining^5–7^. Accordingly, an annual rise of between 10 and 20% on the casting production of these alloys in the last few decades has been reported, and it is projected to maintain at the current rate^8–11^. Aluminum and zinc are alloying elements in magnesium materials, increasing casting and mechanical properties. AZ31 is a commercially available magnesium alloy with excellent casting and mechanical properties^12–15^.

Magnesium composite reinforced by SiC particles has attracted a great attention in various structural fields, from urban transport to airplanes due to its considerable hardness and toughness^8,16–18^. In addition, metal matrix composites (MMCs) show better dimensional stability and abrasion resistance compared to unreinforced alloys. For this reason, these composites have found applications in manufacturing many pistons and valves for automobile engines and are considered for manufacturing aircraft components^19–21^. To produce magnesium-matrix composites reinforced with ceramic particles, several techniques have been frequently used, such as spray deposition, powder metallurgy, squeeze casting, and stir casting. The latter has always been an attractive process due to its simplicity, flexibility, and capability of large quantities production^8,22,23^. Samples with low porosity are achieved in the stir casting method, which is desirable for thermal sink materials^24–28^. Furthermore, stirring the melt during the stir casting causes uniform distribution of reinforcement particles in the matrix and proper bonding of the matrix and the reinforcement. Meanwhile, in the stir casting method, no destruction is caused to the reinforcement particles, which effiectively improves the thermal properties. Because the fracture of reinforcement particles during composite preparation can be a source for crack nucleation during the thermal cycles^29–32^.

Many mechanical parts of car engines and structures used in power plants, petrochemical industries, aerospace, etc., are subject to various mechanical and thermal oscillating loads. Thermal fatigue is the most critical cause of failure in such parts^33–35^. Therefore, it is important to study different methods for assessing thermal fatigue life. When parts are subjected to high-temperature thermal cycles, the thermal fatigue process leads to microstructural damage and eventual component failure^36–38^.

Thermal fatigue, a common type of damage in engineering structures, can be generated in the form of periodic temperature changes, which often occur as a consequence of the complete or partial limitation of thermal deformation^39^. These limitations can be temperature gradient and different thermal expansion attributed to the bonding of different materials^40^. Thermal fatigue can be high-cycle or low-cycle fatigue that depends on the magnitude of thermal stress concerning the yield strength of materials. However, probable mismatch in coefficients of thermal expansion (CTEs) on matrix and reinforcement may cause fluctuations at ambient temperature. It can bring about significant thermal stresses, which may lead to localized plastic deformation in matrix close to reinforcement and instability in dimensions and possible failure in mechanical properties^19,40–42^.

In general, the thermal expansion behavior of composites is the result of several different parameters of materials e.g., compounds, phases, reinforcement content, and the distribution of reinforcement in the matrix^42^. Thermal cycle experiments in a specific temperature range can simulate and evaluate their thermal fatigue behavior^39,43,44^. Thermal fatigue test is performed in different ways. Three essential parameters determine the operating conditions of the cycle, which include the maximum temperature, the amount of strain, and the time per cycle. Generally, the difference between these methods is in the way of cooling and heating^43^.

Since Mg/SiC_p_ composites are used for engineering parts when subjecting subjected to thermal cycles, resulting in thermal stress and microthermal cracks at the interface between reinforcement and matrix. Having a good knowledge on thermal fatigue characteristics of these composites during thermal variation is essential.

Nowadays, car manufacturers are developing new technologies to replace aluminum materials with magnesium materials. There have been numerous studies about the thermal properties of aluminum composite^45–47^. However, less research has been conducted on the behavior of thermal fatigue in magnesium-based reinforced composites compared with that of the aluminum-matrix composites. Zhao et al.^17^ reported on how the thermal cycle may affect the mechanical properties of ZK60/SiC composite produced by squeeze casting and concluded that changes in matrix microstructure after the thermal cycle is critical in the mechanical properties of ZK60/SiC composite. During a thermal cycle, the tensile strength of the ZK60/SiC composite declined due to cyclic thermal stresses in the matrix of the composite. Huang et al.^41^ studied the effects of composition and orientation of fiber and then heat treatment on the thermal strain of KS1275 and AE42 alloys and composites. Their results showed that thermal strain changed slightly throughout the thermal cycle after aging. Moreover, heat treatment led to an increase in matrix yield stress. Furthermore, thermal stress was reduced mainly by matrix plastic deformation preferred than by interfaces debonding. Pan et al.^39^, who researched nucleation and propagation behavior of composite thermal fatigue cracks produced by stir casting were studied at 250 °C and 150 cycles, drew a conclusion that particle distribution was a contributing factor in preventing propagation of thermal fatigue cracks; hardness values of composites decreased with an increase in a number of cycles.

Notwithstanding, there is still little research about thermal fatigue behavior in magnesium-based composites reinforced with SiC particles. Features of the thermal crack growth process have yet to be studied, the addition of SiC reinforcing particles with lower CTE and higher thermal conductivity than AZ31 matrix alloy possibly led to the fabrication of AZ31/SiC composites with low CTE and higher thermal conductivity and subsequently higher thermal fatigue resistance^48^.

For this purpose, thermal cycling behavior concerning AZ31 alloy and AZ31 reinforced with 5 and 10 wt% SiC, as well as nucleation and propagation of thermal fatigue cracking under thermal cycle from room temperature (RT) to 150 and 350 °C for 300, 600, and 900 heating and cooling cycles were investigated.

## Materials and method

### Materials

To prepare the AZ31/SiC composite, SiC particles (40 μm) with high purity as a reinforcement and Mg-Zn, and Mg–Al master alloys were used to prepare the AZ31 alloy as a matrix.

### Sample preparation

Melting, alloying, and compositing were performed at the induction furnace at 780 °C. The alloying process was performed by melting magnesium and adding Mg-Zn and Mg–Al master alloys to the specified amount. The prepared alloy melt was then cast into steel cylinder molds, and ICP-OES was used to determine the chemical composition. The chemical composition of the base alloy is presented in Table 1.

**Table 1:** The chemical composition of the AZ31 alloy.

Elements	Al	Zn	Mg
wt.%	3	1	Bal

The stir casting process was used to produce the AZ31/SiC composites. After alloying, 5 and 10 wt% preheated SiC was added to the melt and stirred with a titanium stirrer for 10 min. Composite ingots were cast in steel molds. CO_2_ + SF_6_ gas was used as a protection to prevent oxidation of the melt. The detail of sample preparation is described in^1^.

To investigate the effect of precipitation hardening on the thermal properties of the samples, the samples were subjected to dissolution at 400 °C for 3 h, subsequently quenched in water, and then aging at 180 °C for 6 h^49^.

**Figure 1:**
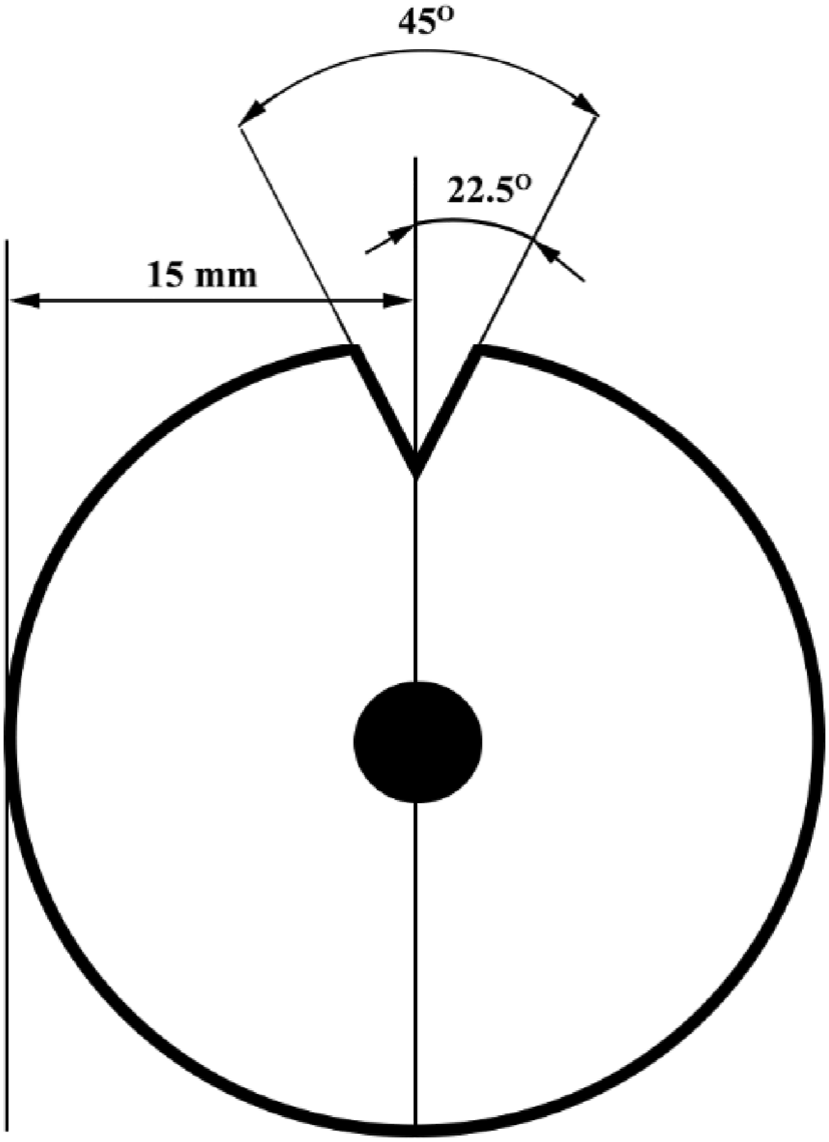
Schematic of sample for thermal cycles.

Alloy and composite samples were cut with dimensions of 30 φ × 5 mm^3^, and then a V-shaped notch was cut with an angle of 45° and a depth of 2 mm, according to Fig. 1.

### Density

Archimedes' method^50^ was used to calculate the porosity and density of the samples.

### Hardness

For hardness measurement based on ASTM E10, the Wolpert microVickers method was employed under 500 g force for 20 s. In all samples, five distinct points were measured, and the erroe bar was calculated and reported.

### Thermal test

#### Dilatometry

The coefficient of thermal expansion of AZ31 alloy and AZ31/5% SiC and AZ31/10% SiC composites were measured by a BAEHR dilatometry device (Model DIL 801, German) in the RT to 350 °C range with cooling and heating rates of 5 °C/min.

#### Thermal cycle test

**Figure 2:**
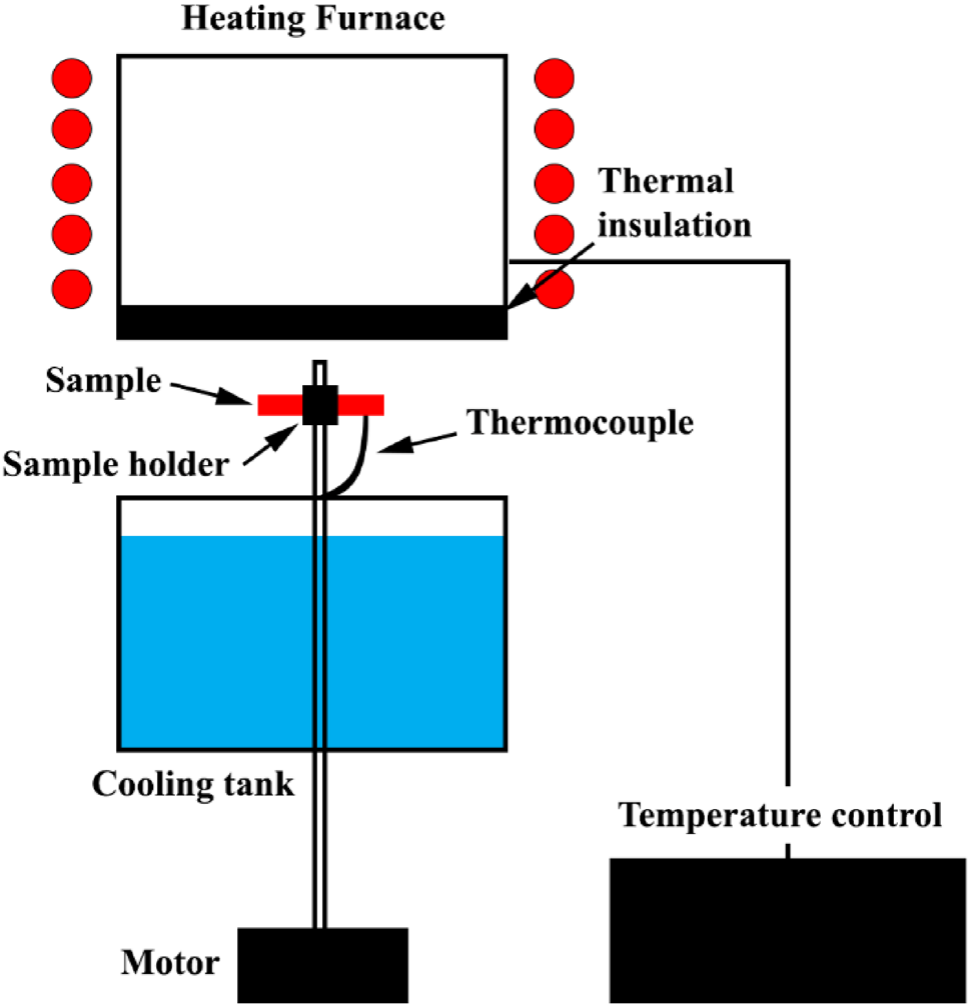
Schematic of setup of thermal cycles.

**Figure 3:**
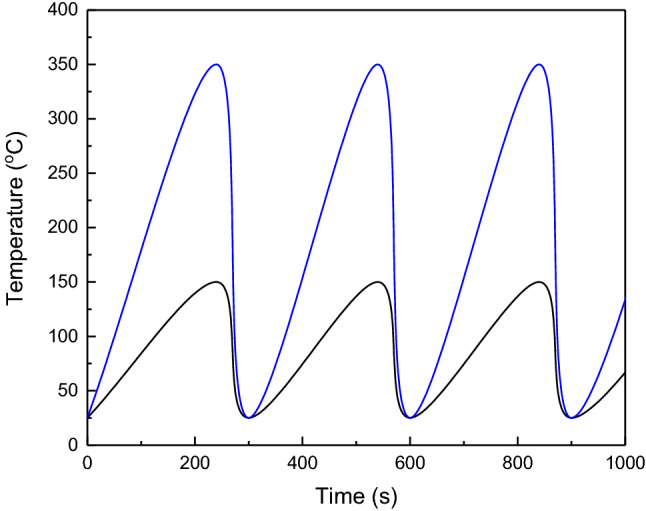
Thermal cycles at 150 and 350 °C versus time.

To investigate the thermal cycles behavior, the sample was mounted on a movable lever. The lever can transfer the sample from the furnace to the cooling tank and vice versa. Moreover, to control the sample temperature accurately, a thermocouple was attached to the sample and the samples were heated in an electric furnace (Model VM16L-1200), which was insulated with refractory blanket. Figure 2 shows the schematic of the thermal fatigue setup. Alloy and composite samples were undergone 300, 600, and 900 heating/cooling cycles were applied at 150 °C and 350 °C. The soaking times for the heating and cooling cycles were 4 and 1 min, respectively, and water was used as the cooling medium. Figure 3 displays the thermal cycles graphs.

### Characterizations

The Philips X-ray diffraction (XRD) analysis was used to investigate the formed phases. The device's voltage was 40 kV, and the applied current was 30 mA. A copper cathode X-ray tube with a wavelength of 1.54059 Å was used. The scanning step size was selected as 0.05° in the range of 10 to 90°. The X’pert HighScore software version 3.0 was then used to analyze the data.

Cutting the samples using a large amount of cooling lubricant prevented heat generation, thus the possibility of affecting the microscopic structure of the samples. The samples were then ground with numbers 200, 400, 600, 800, 1000, 1500, 2000, and 2500 sandpaper sheets according to the European FEPA system and then polished with alcohol and diamond paste. A solution (6 g of pyric acid, 5 ml of acetic acid, 10 ml of distilled water, and 100 mL of ethanol) was used to reveal the microstructure. Microstructure observation was performed using the IMM-420 optical microscope (OM) and the VEGA3 LMU scanning electron microscope (SEM) equipped with energy dispersive X-ray spectroscopy (EDS). The CL was measured as per ASTM E 647–99 protocol.

## Results and discussion

### Microstructure

**Figure 4:**
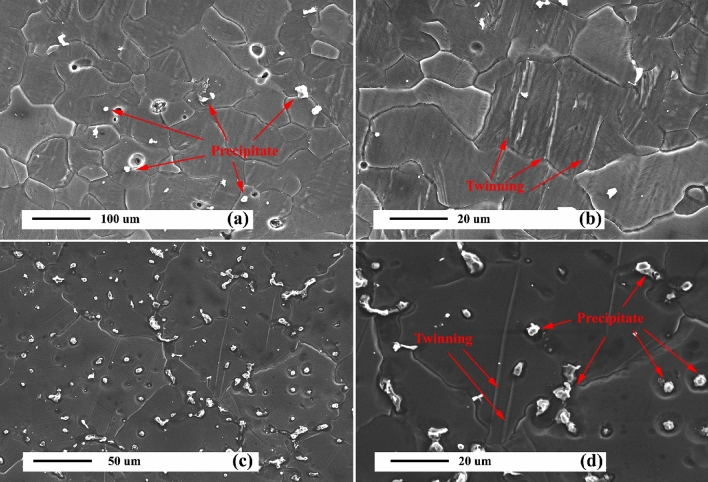
SEM micrographs of unreinforced alloy: (**a**) and (**b**) as-cast, and (**c**) and (**d**) after precipitation hardening.

Figures 4a and b reveal the SEM micrographs of the as-cast AZ31 alloy. As can be seen, the AZ31 alloy consists of coaxial grains with an average grain size of 100 μm. The spherical coarse and discrete residual phases in the microstructure of as-cast AZ31 alloy is especially evident in the grain boundaries, which is the Mg_17_Al_12_ phase, according to the previous study^1^. The Mg_17_Al_12_ phase is formed during solidification due to segregation and the phase characteristic of a low melting point. In addition, some twinning can be seen in the matrix, which are identified by the arrow in Fig. 4b. The formation of twins depends on the crystal structure of the Mg alloy. Mg alloys typically have a hexagonal structure (HCP) that fails to provide five independent slip systems to meet the Von Mises standard for uniform plastic deformation at RT. Also, the plastic deformation in directions a and c in the HCP structure is anisotropic. These properties cause the activation energy of twinning deformation to have less slip on the prismatic and pyramidal planes^51^. Therefore, in the Mg alloy, it generates the energy required to form the twins at low temperatures. Figure 4c and d demonstrate the SEM micrographs of the aged AZ31 alloy. The grain size is equal to 80 μm, which is 20% smaller than that of the as-cast alloy. The uniform presence of precipitates is observed throughout the microstructure. Furthermore, a limited number of thermal twins are observed in the as-cast AZ31 alloy microstructure.

**Figure 5:**
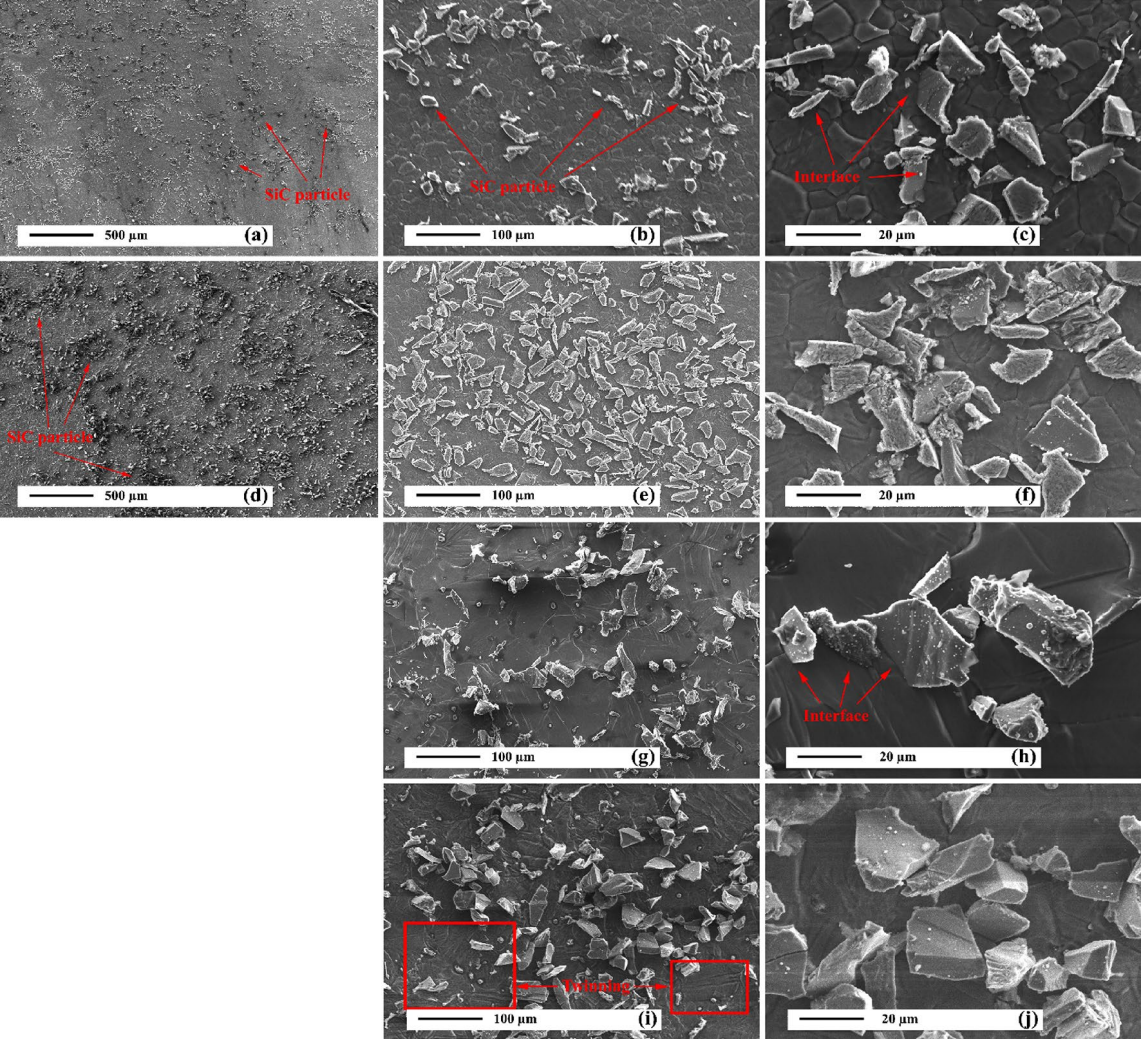
SEM micrographs of composite samples: (**a**, **b**, and **c**) as-cast AZ31/5%SiC, (**d**, **e**, and **f**) as-cast AZ31/10%SiC, (**g**, and **h**) AZ31/5%SiC after precipitation hardening, and (**i**, and **j**) AZ31/10%SiC after precipitation hardening.

Figure 5a–f show the SEM micrographs of the AZ31/5%SiC and AZ31/10%SiC as-cast composites. Relatively homogeneous distribution of SiC particles is seen in the AZ31 matrix. In the higher magnification micrograph (Fig. 5c,f), a matrix/reinforcement interface free of cracks, porosity, and cavities can be seen, which indicates good wettability of SiC particles by Mg melt. The average grain sizes of AZ31/5%SiC and AZ31/10%SiC composites were 30 and 20 μm, respectively. The smaller grain size of the composites compared to the unreinforced alloy is related to the presence of SiC particles and the heterogenic solidification on the surface of the SiC particles, which has caused the refinement^52–54^. However, due to the refinement of the matrix, the number of twins is less than those in the unreinforced sample.

Figures 5g–j reveal the SEM micrographs of the AZ31/5%SiC and AZ31/10%SiC aged composites. The relative homogeneous distribution of reinforcement particles is observed in both composites, obtained without any porosity and cracks. The average grain sizes of AZ31/5%SiC and AZ31/10%SiC composites were 16 μm and 10 μm, respectively. Precipitation hardening and increasing the percentage of reinforcement particles caused the grain size to reduce significantly^55^. By decreasing the grain size, the activation energy required to form twins increases^56^. However, Fig. 5i shows the presence of twins in the microstructure, created in aging treatment. As shown in the higher magnification micrographs in Fig. 5h,j, in AZ31/5%SiC and AZ31/10%SiC composites, proper adhesion is created between the reinforcing particles.

### XRD

**Figure 6:**
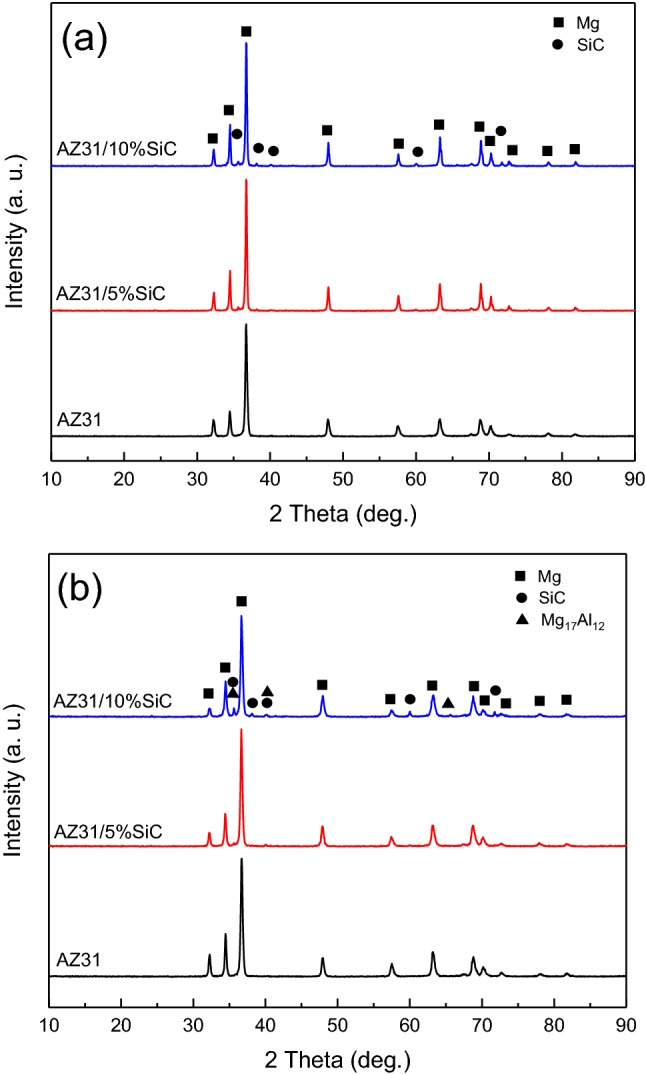
XRD patterns of samples: (**a**) as-cast, and (**b**) after precipitation hardening.

XRD patterns prepared from AZ31, AZ31/5%SiC, and AZ31/10%SiC samples with as-cast and aged conditions are shown in Fig. 6. As seen in Fig. 6a, the peaks of the Mg alloy and SiC are quite evident, and no unwanted carbide phases were observed. Also, for aged samples in Fig. 6b, no interaction was observed between SiC and Mg, which could create significant undesirable phases during the precipitation hardening. However, Mg_17_Al_12_ precipices are detected in Fig. 6b.

### Density

**Figure 7:**
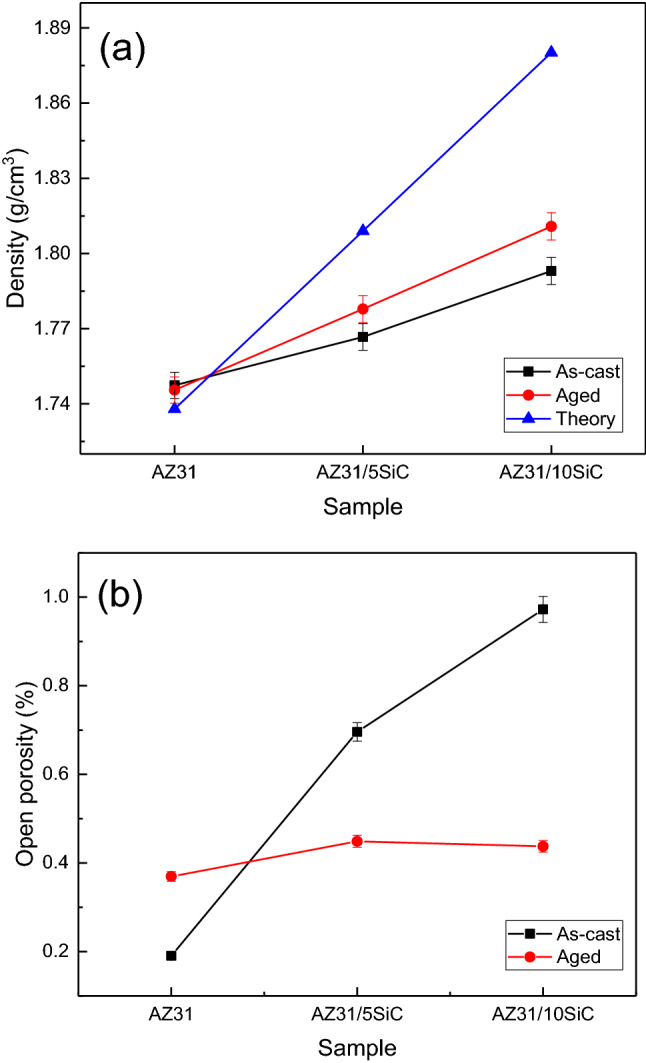
(**a**) Density of as-cast and aged samples, and (**b**) open porosity of as-cast and aged samples.

The density and open porosity of the as-cast samples were measured by the Archimedes method and given in Fig. 7. The density of AZ31 alloy is almost the same compared to the aged state. The experimental density is slightly different from the theoretical density, which indicates good matrix/reinforcement interface adhesion and low porosity in the samples. With increasing reinforcement content, the porosity and density increased due to the high density of reinforcement particles. Based on Fig. 7b, it is clear that the open porosity has increased with increasing reinforcement percentage. The increase in porosity with increasing reinforcement particles can be related to the partial agglomeration between the particles during stirring in the casting mode, which increases the porosity. These porosities are due to the production process, and their presence in the production process is inevitable. By comparing the open porosity diagrams in the as-cast and aged samples, it is clear that the porosity of the aged samples has decreased, which is related to the closure of cavities at high temperatures.

### CTE

**Figure 8:**
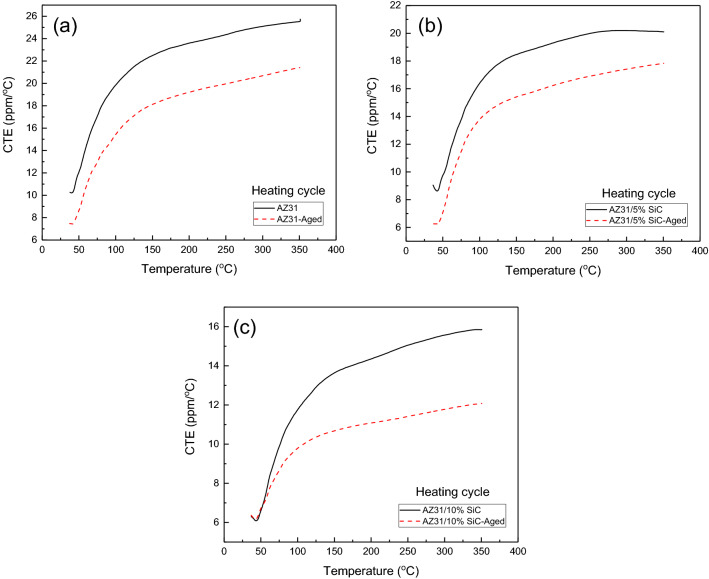
CTE variation verse temperature for as-cast and aged samples: (**a**) unreinforced alloy, (**b**) AZ31/5%SiC, and (**c**) AZ31/10%SiC.

Figure 8 shows the CTE changes in terms of temperature for as-cast and aged samples of AZ31 alloy and AZ31/SiC composites in the heating and cooling cycles. As can be seen, the CTE for the as-cast AZ31 in the heating cycle up to 350 °C is close to 26 ppm/°C and for the AZ31/5%SiC and AZ31/10%SiC samples is close to 20 and 15 ppm/°C, respectively. The reduction of CTE for composite samples is related to lower CTE of SiC (4 ppm/°C). The results show that in the cooling cycle starting from 350 °C, a hysteresis is created in the CTE diagram. Also, the changes in the CTE in the cooling cycle for the as-cast and aged samples are almost close to each other because the strains created in the casting process are released at 350 °C. The CTE for the aged AZ31 in the heating cycle up to 350 °C is close to 22 ppm/°C and for the aged AZ31/5%SiC and AZ31/10%SiC samples is close to 18 and 12 ppm/°C, respectively, which shows 15%, 10%, and 20% reduction of CTE of aged samples compared to the as-cast samples, respectively. This reduction is related to the refinement of the aged samples compared to the as-cast samples. Also, the difference between as-cast CTE and aged CTE samples in alloys is more than in composite samples because the aging process is more effective than in refines in alloy compared to the composite samples (Figs. 4 and 5). Based on the previous study^57^, in the first heating cycle, the maximum plastic deformation occurs, and residual strain changes in the first cycle were examined by dilatometry. The AZ31 alloy was not expected to contain hysteresis due to the high residual strain due to the absence of ceramic particles in the microstructure.

Hysteresis was evident in the unreinforced alloy, as seen in the thermal cycling graphs in Fig. 8. Regarding composites samples, compressive plastic deformation was caused by internal thermal mismatch stresses of matrix/reinforcement and started at ~ 150 °C, as shown by a reduction in instantaneous CTE values. This led to create hysteresis due to the residual strain during the thermal cycling. Plus, the temperature at that plastic deformation starts for AZ31/10%SiC is the same as that observed earlier for AZ31/5%SiC^57,58^. The internal thermal stresses induced by a temperature change (ΔT) in a composite sample, are given by^59^.

1 $$ \sigma_{TS} = \frac{{E_{r} E_{m} }}{{E_{r} f + E_{m} \left( {1 - f} \right)}}f\Delta \alpha \Delta T $$

where $E_{m}$ and $E_{r}$ are Young’s moduli of the matrix and reinforcement, respectively, f is the reinforcement fraction, and $\Delta \alpha$ is the matrix and reinforcement CTEs differences.

Based on calculations, 150 °C was the temperature justified for plastic deformation in AZ31/5%SiC^57,60^. Similarly, in this research, plastic deformation of AZ31/5%SiC is expected to occur at this temperature. Using particulate ceramic reinforcement, the occurrence of global matrix yielding by the dislocation glide is not probable in the hydrostatic stress field around the reinforcement particle. However, in every local region, thermal stresses are not hydrostatic, and the plastic deformation can occur by a generation of the mobile secondary dislocation^61–63^. Rudajevova et al.^64^ have demonstrated that plastic deformation in QE22/15–25%SiC composite takes place at 240 °C and 255 °C, respectively. Since AZ31 has higher plastic deformation than SiC, greater residual strain and hysteresis are very probable, as seen in Fig. 8.

### Thermal cycles

#### As-cast

##### Microstructure

**Figure 9:**
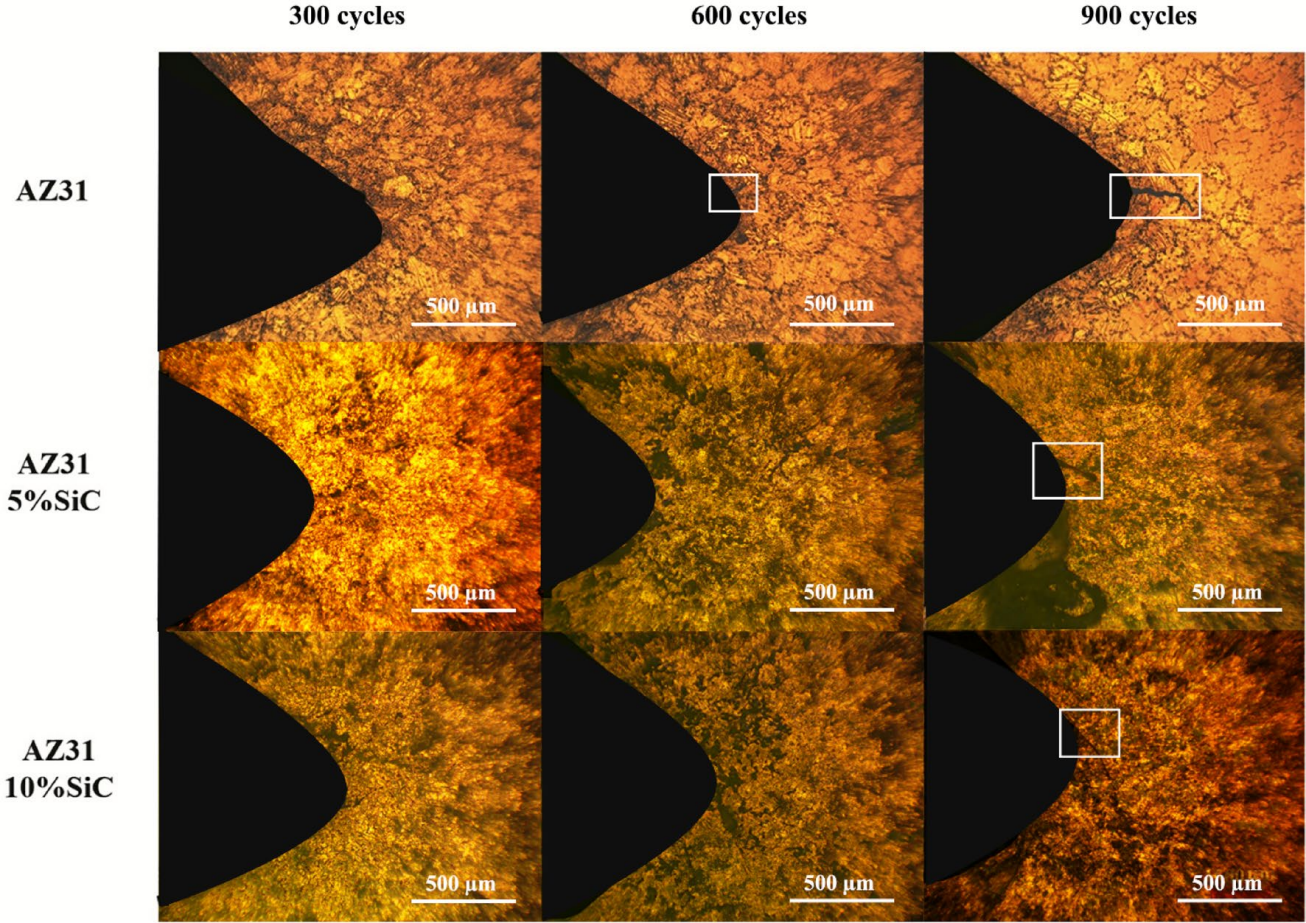
OM micrographs of as-cast unreinforced alloy and composites samples after 300, 600 and 900 thermal cycles at 150 °C.

**Figure 10:**
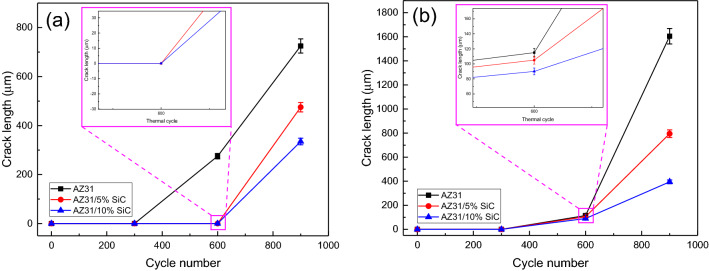
Crack length of the as-cast samples after thermal cycle at: (**a**) 150 °C, and (**b**) 350 °C.

Figures 9 and 10a reveal the OM micrographs of the notch tip of the as-cast samples and quantitative measurement of the CLs under different thermal cycles at 150 °C, respectively. It can be seen that no cracks were nucleated after 300 cycles in the unreinforced alloy, but it suffered a CL of 275 μm after 600 cycles, and by increasing the number of cycles to 900, the CL increased to 725 μm, which indicates a rise of 450%. Meanwhile, in a previous study^65^ on the thermal fatigue of AZ91 alloy containing RE up to 400 thermal cycles have been recorded before the failure of samples at temperatures of 170 and 210 °C. By comparing their results to the ones of this study, it can be seen that the thermal fatigue resistance of their alloy was much lower than that of the present studied alloy.

**Figure 11:**
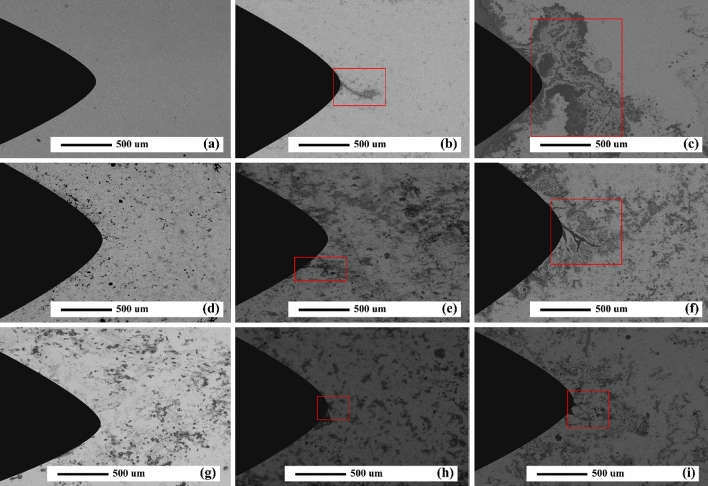
SEM micrographs of as-cast samples after thermal cycles at 350 °C: (**a**) unreinforced alloy after 300 cycles, (**b**) unreinforced alloy after 600 cycles, (**c**) unreinforced alloy after 900 cycles, (**d**) AZ31/5%SiC after 300 cycles, (**e**) AZ31/5%SiC after 600 cycles, (**f**) AZ31/5%SiC after 300 cycles, (**g**) AZ31/10%SiC after 300 cycles, (**h**) AZ31/10%SiC after 600 cycles, and (**i**) AZ31/10%SiC after 900 cycles.

In AZ31/5%SiC and AZ31/10%SiC samples, after 900 cycles, CL is 35% and 50% shorter than that of unreinforced alloy with the same number of cycles, respectively. SEM micrographs of the notch tip of the as-cast samples and quantitative measurement of the CLs under thermal cycle at 350 °C are shown in Figs. 10b and 11, respectively. According to the Fig. 11, no crack is observed after 300 cycles, and a 115 μm long crack is observed in the unreinforced AZ31 alloy after 600 cycles and with increasing the number of cycles, CL grows and reaches 1605 μm at 900 cycles, which shows an increase of 1490%. Conditions for AZ31/5%SiC and AZ31/10%SiC samples are as follows: in 300 cycles, no crack is left at the tip of the notch, and after 600 cycles, 105 and 90 μm cracks are observed, respectively, which is similar to the crack with the same number of cycles for the unreinforced alloy is 9% and 22% reduction, respectively. Also, for AZ31/5%SiC and AZ31/10%SiC samples in 900 cycles, 795 and 395 μm cracks are evident, respectively, which is 50% and 75% less than cracks with the same number of cycles of the unreinforced alloy. It was observed that the CLs at 350 °C were increased compared to thermal cycles at 150 °C. With an increase in cycle times, the interfacial strength of composites experienced a decline, and cracks grew along low-strength areas of the composite where there is a random distribution of particles and micro cracks. At low thermal cycles, crack growth may experience deviation for the composite while crack growth of unreinforced alloy is straight. Furthermore, a couple of factors impact the growth path of a thermal fatigue crack, and crack morphology varies as heating cycles and cooling cycles change. Since matrix and particle have a great diversity of CTE, at their interfaces, there will possibly be thermal stresses when cycles changes; they influence composite strength, thus reducing the mechanical properties of composite materials. By comparing the experimental results with the ones reported by Russell-Stevens^66^ regarding the thermal fatigue behavior of AZ91D/60% carbon fibre composites in the temperature range of 100 to -100 °C for up to 100 cycles, it is evident that the presence of SiC ceramic particles has a greater effect on the thermal fatigue resistance.

**Figure 12:**
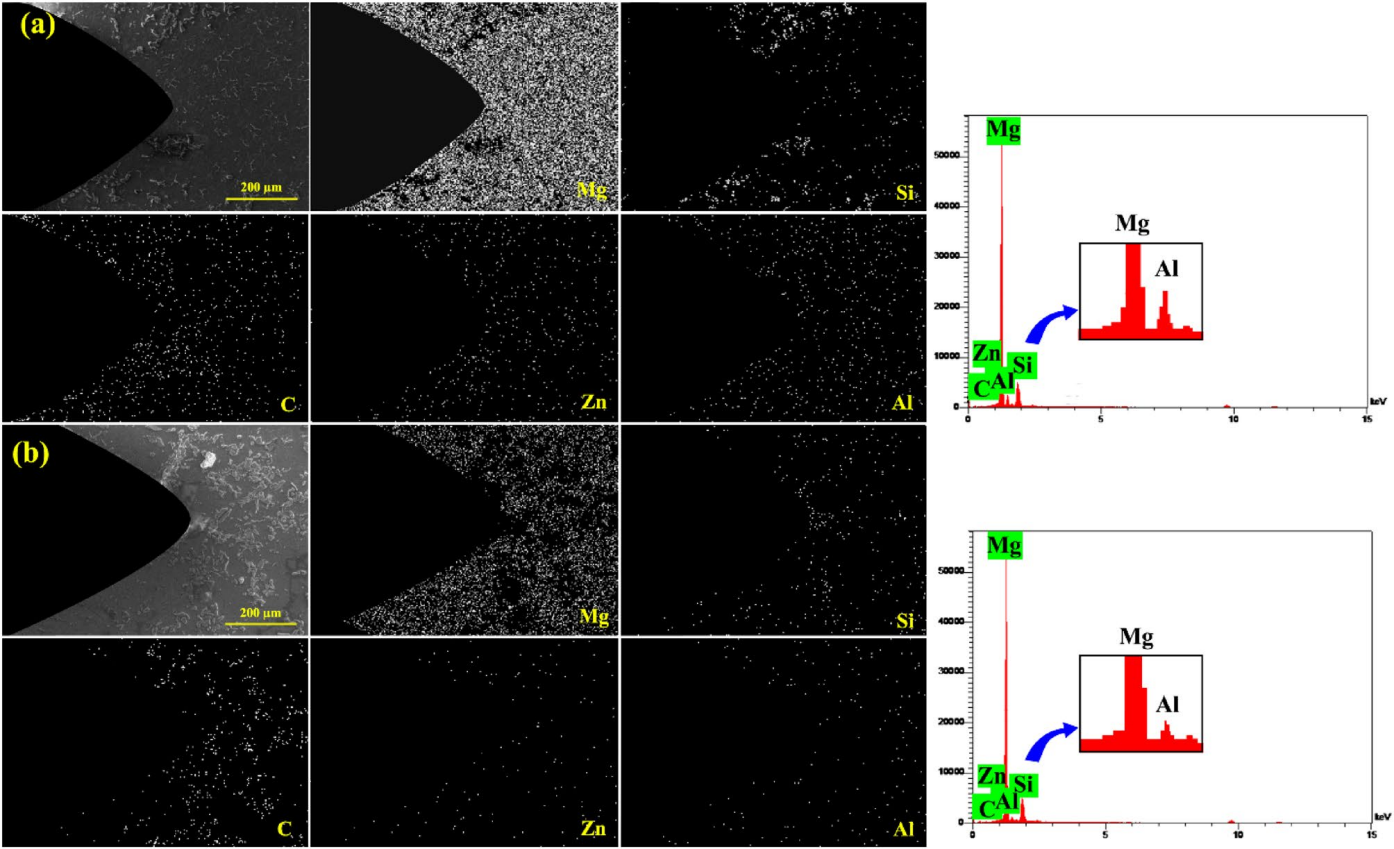
Elemental map analysis of the sample: (**a**) before thermal cycle, and (**b**) after 300 thermal cycles at 350 °C.

Figure 12 shows the elemental map analysis of as-cast AZ31/10%SiC composite before and after 300 heating and cooling cycles at 350 °C. It is observed that the distribution of aluminum elements at the edge of the notch and the crack site is less than in the matrix. In addition, silicon elements was also observed at the crack site. Therefore, the diffusion of aluminum and the accumulation of silicon at the crack site contributed to the growth of cracks.

##### Density

**Figure 13:**
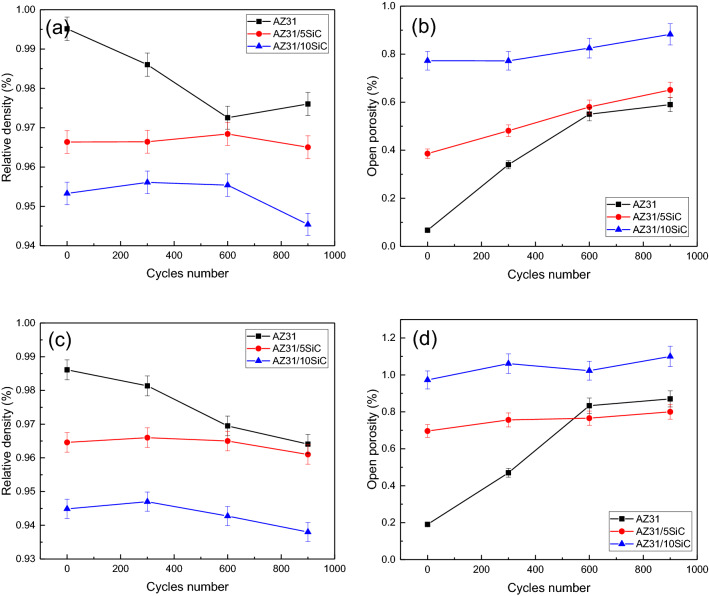
(**a**) Relative density of as-cast samples after thermal cycles at 150 °C, (**b**) open porosity of as-cast samples after thermal cycles at 150 °C, (**c**) relative density of as-cast samples after thermal cycles at 350 °C, and (**d**) open porosity of as-cast samples after thermal cycles at 350 °C.

Figure 13a shows the density changes in terms of the number of cycles at 150 °C of unreinforced alloy and AZ31/5%SiC and AZ31/10%SiC composites. According to the figure, it is clear that before performing the thermal cycle, the alloy sample has the highest density, followed by the AZ31/5%SiC sample, and the lowest density is related to AZ31/10%SiC sample. This can be related to the existence of primary cavities in the microstructure of the composites. Preparation of a composite with a high percentage of reinforcement but without creating a cavity is inevitable. As the number of cycles increases to the first 600, the density changes for composite samples are almost constant. However, the density of unreinforced alloy decreased significantly with increasing the number of cycles up to 600. In the open porosity graph in Fig. 13b, the relationship between porosity and the number of cycles at 150 °C for unreinforced alloy and AZ31/5%SiC and AZ31/10%SiC composites can be examined. By increasing the number of cycles up to the first 600, the open porosity changes for composite samples are almost constant. This indicates that at 150 °C, the energy required for the growth of cracks in the microstructure and the energy required for the nucleation of new cracks for the composites is not provided, causing a new porosity. However, the unreinforced alloy sample has a marked increase in porosity by increasing the number of cycles to 600.

In fact, for the alloy sample, the energy required for crack nucleation and crack growth is provided. Porosity is an intrinsic property of the composite. However, during thermal cycle testing, cracks can be optimally formed from these porosities^67^. Figure 13c and d show a graph of density changes and porosity percentages in terms of the number of cycles at 350 °C of unreinforced alloy and AZ31/5%SiC and AZ31/10%SiC composites. The conditions for as-cast samples under thermal cycles of high temperature (350 °C) are similar to those of low temperature (150 °C). However, the AZ31/5%SiC sample also showed an increase in open porosity at 350 °C from 300 to 600 cycles, which is the reason for the increase and growth of cracks. No noticeable change is observed in most thermal cycles up to 900 cycles.

#### Aging

##### Microstructure

**Figure 14:**
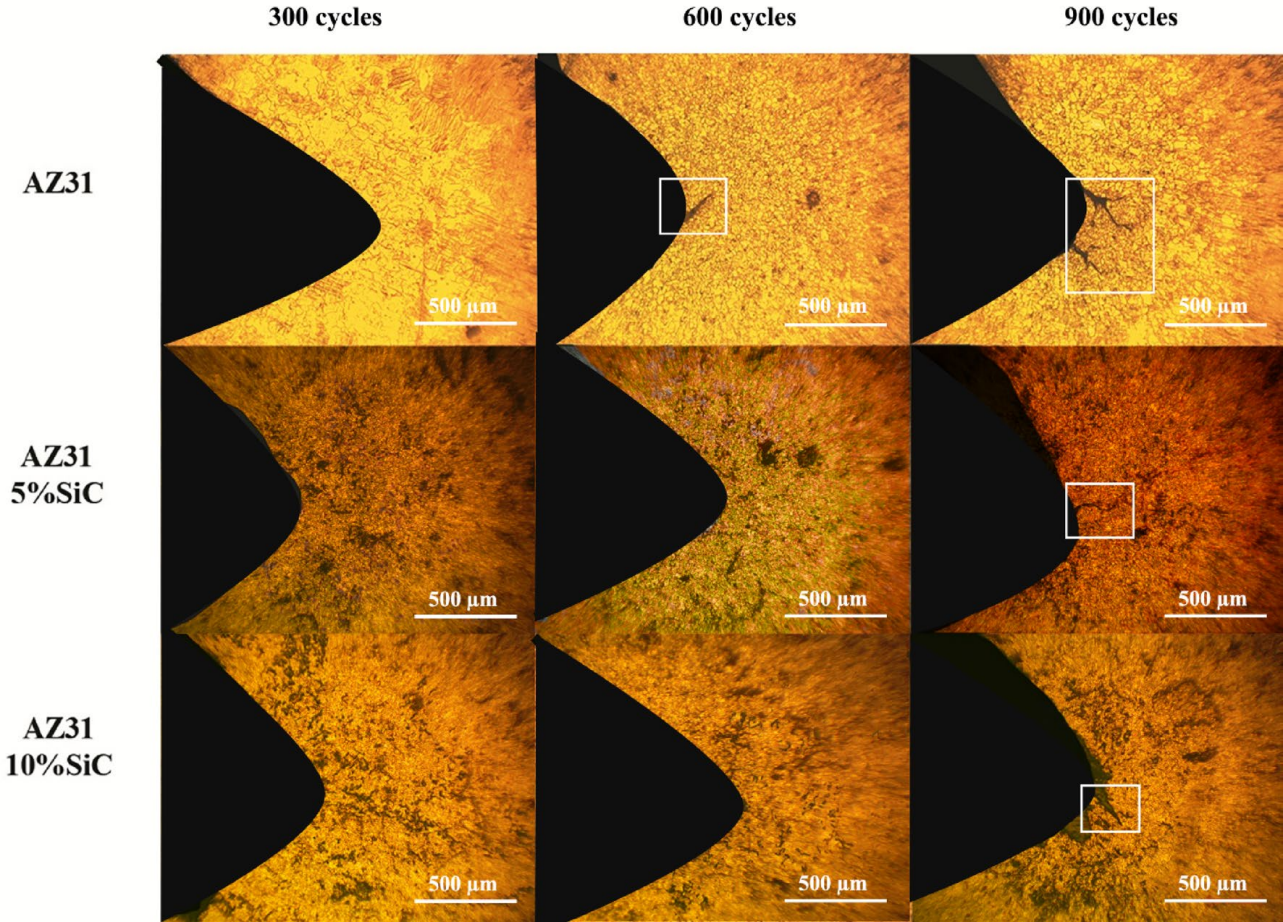
OM micrographs of aged unreinforced alloy and composites samples after 300, 600 and 900 thermal cycles at 150 °C.

**Figure 15:**
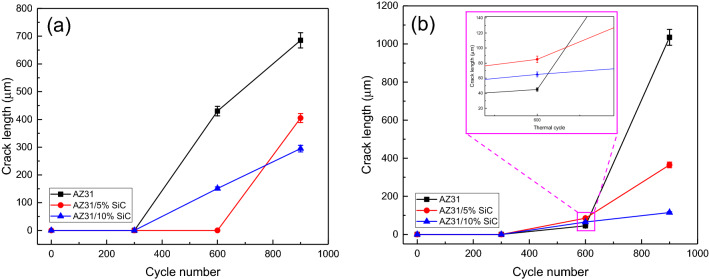
Crack length of the aged samples after thermal cycle at: (**a**) 150 °C, and (**b**) 350 °C.

**Figure 16:**
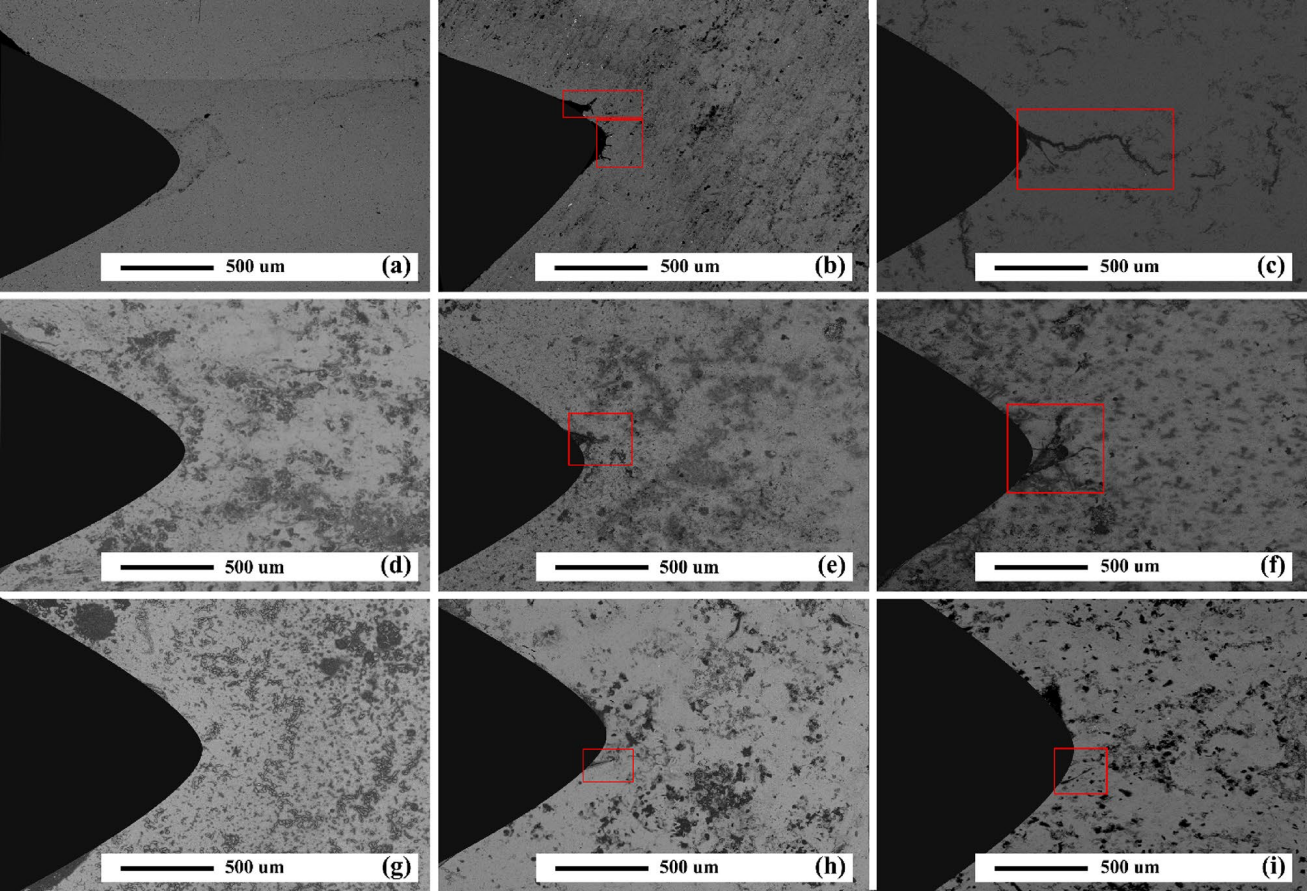
SEM micrographs of aged samples after thermal cycles at 350 °C: (**a**) unreinforced alloy after 300 cycles, (**b**) unreinforced alloy after 600 cycles, (**c**) unreinforced alloy after 900 cycles, (**d**) AZ31/5%SiC after 300 cycles, (**e**) AZ31/5%SiC after 600 cycles, (**f**) AZ31/5%SiC after 300 cycles, (**g**) AZ31/10%SiC after 300 cycles, (**h**) AZ31/10%SiC after 600 cycles, and (**i**) AZ31/10%SiC after 900 cycles.

Figures 14 and 15a demonstrate the OM micrographs of the notch tip of aged samples and quantitative measurement of the CLs under different thermal cycles at 150 °C, respectively. Based on the Fig. 14, CL reached 685 μm after 900 cycles, which shows a growth of 60% compared to the as-cast sample. After 900 cycles, AZ31/5%SiC and AZ31/10%SiC samples showed crack nucleation and growth, 40% and 60% less than the unreinforced alloy. SEM micrographs of the notch tip of the aged samples and quantitative measurement of the CLs under thermal cycles at 350 °C are shown in Figs. 15b and 16, respectively. According to the Fig. 16, it is clear that after 600 cycles, the unreinforced AZ31 alloy cracks nucleated and grew to a length of 45 μm, and by increasing the number of cycles to 900, the crack grew and reached a length of 1035 μm, showing an increase of 2200%. After 300 cycles, no crack was observed at the notch tip for AZ31/5%SiC and AZ31/10%SiC, and after 600 cycles, 85 μm and 65 μm long cracks were formed, respectively. Furthermore, after 900 cycles, 365 and 115 μm long cracks were observed for AZ31/5%SiC and AZ31/10%SiC samples, 65% and 88% less than unreinforced alloy cracks with the same number of cycles, respectively.

During the entire process, cracks may meander. This is because of particles that are likely to affect the direction of the crack tip, and then cracks move forward along less strong positions, namely voids and weak interfaces. Noteworthy is the fact that bonding strength concerning the interface of particle and matrix may impact the stress intensity factor at the crack tip. In addition, when the main crack propagated, a distinct pattern of crack appeared at the crack front. It is believed that detached particles or void defects are responsible for this pattern's occurrence; in fact, it seems they merge into the main crack, thus accelerating crack growth. Figure 16 displays the front of the main crack where sub-cracks were configurated and combined after several cycles. Defects and any areas like Mg, SiC particles, and interface where strength is low are favorable sites in which crack may initiate and then propagate directionally.

##### Density

**Figure 17:**
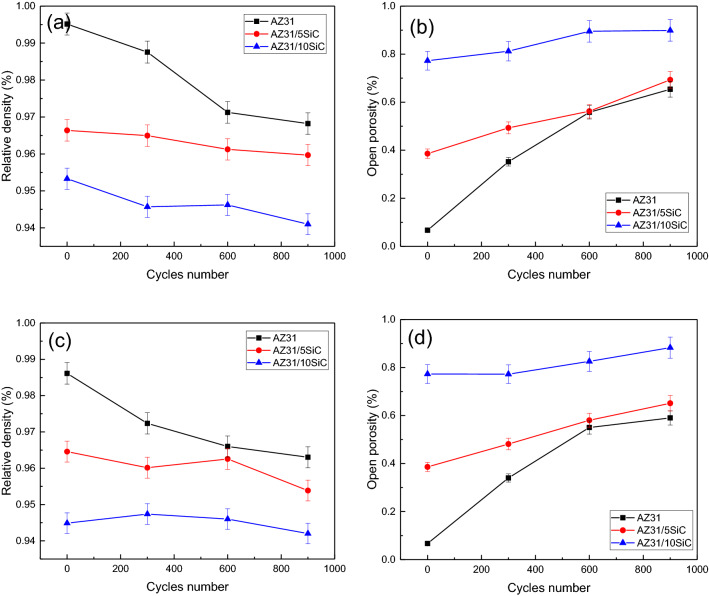
(**a**) Relative density of aged samples after thermal cycles at 150 °C, (**b**) open porosity of aged samples after thermal cycles at 150 °C, (**c**) relative density of aged samples after thermal cycles at 350 °C, and (**d**) open porosity of aged samples after thermal cycles at 350 °C.

Figure 17a depicts the density changes in terms of cycle numbers at 150 °C for unreinforced alloy and AZ31/5%SiC and AZ31/10%SiC composites. As the number of cycles increases to the first 600 cycles, the density changes for composite samples are almost constant. This indicates that at 150 °C, the activation energy required for the nucleation/growth of cracks in the microstructure is not provided, causing changes in density. However, the density of the unreinforced alloy sample decreased significantly with an increasing number of cycles up to 600. In fact, for the unreinforced alloy sample, the energy required for crack nucleation and crack growth is provided. From open porosity diagrams in Fig. 17b, the relationship between porosity and the number of cycles can be examined for unreinforced alloy and AZ31/5%SiC and AZ31%10%SiC composites at 150 °C. By increasing the number of cycles up to 600, the open porosity changes for composite samples are almost constant. This indicates that at 150 °C, the energy required for the growth of cracks in the microstructure and the energy required for the nucleation of new cracks for the composites is not provided, causing new porosity to form. However, the unreinforced alloy sample has a marked increase in porosity by increasing the number of cycles to 600. The activation energy required for crack nucleation and crack growth is provided for the alloy sample.

Figure 17c and d show the density and open porosity percentage changes in the a number of cycles at 350 °C for unreinforced alloy and AZ31/5%SiC and AZ31%10%SiC composites. Conditions for aged samples under high temperature (350 °C) thermal cycles are similar to those of low temperature (150 °C). As the precipitates created in the microstructure, the amount of density and porosity in aged samples under thermal cycles increased and decreased compared to as-cast samples. Furthermore, no MgO was detected at the interface during thermal cycling. It is well-known that 350 °C for long periods is presumably required for carbide formation in Mg/C composites^68^. The upper temperature of 100 °C and short pauses involved in the present study are not high enough to activate interfacial reactions. Since the chemical state of the interface remains unchanged, composite flexural strengths do not depend on thermal cycling, and when there is no chemical degradation during the thermal cycle, mechanical damage of interfacial is bound to occur. After thermal cycling, this mechanical damage appeared in the form of microvoids, microcracks, and particle protrusion triggered by interfacial sliding.

**Figure 18:**
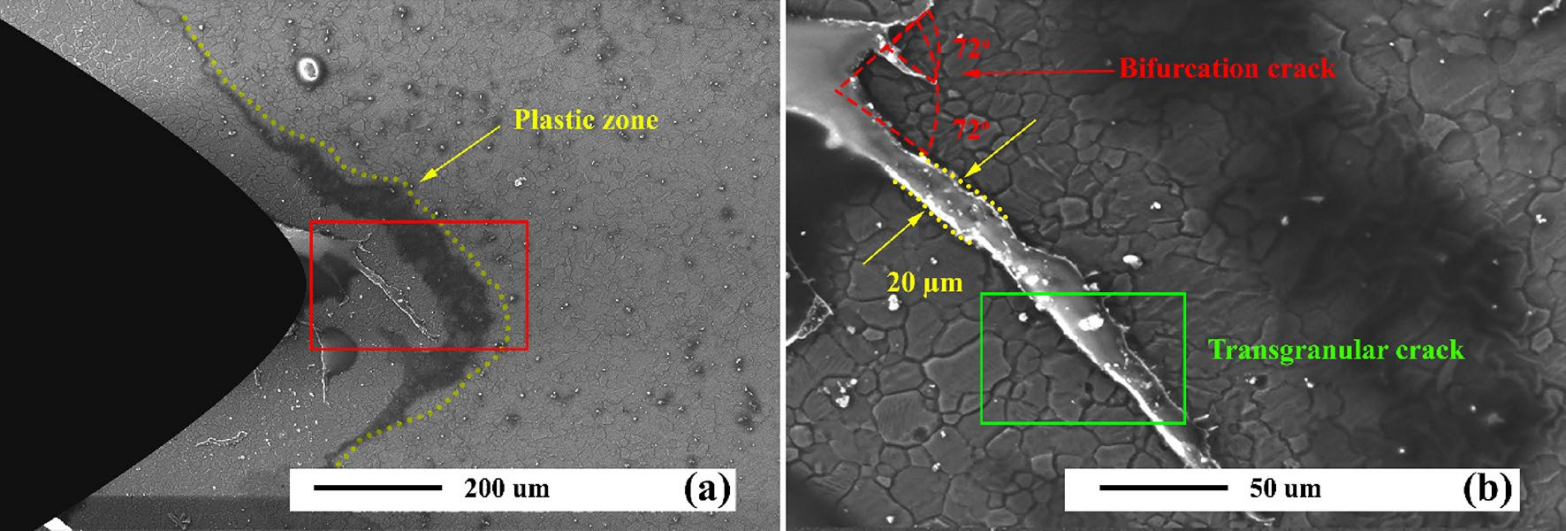
(**a**) SEM micrograph of aged unreinforced alloy after thermal cycles at 150 °C, and (**b**) higher magnification.

Interfacial micro-cracking and void formation are evident in composites after 300 cycles (Fig. 18), and neither of them seemed to get worse with a rise in the number of thermal cycle. However, there is not any quantitative study. Therefore, it is believed that sample preparation is often responsible for the occurrence of microcracking. Nevertheless, despite many efforts to prevent the creation of preparation artifacts, they were still unavoidably poorly bonded composites. Microvoid formation has been frequently detected in Cu matrix composite during the thermal cycles^69^. Microcracks and microvoids are likely to weaken reinforcement/matrix interfacial bond, and load may be transferred to the reinforcement phase. As can be seen, particle protrusion took place after thermal cycling in AZ31/SiC, as a result of interfacial sliding during cooling cycles wherein particle expands in matrix contracts and longitudinal direction.

Nonetheless, it is also possible that sliding takes place during the whole thermal cycle which is not limited to cooling cycle. In present study, composites samples have been evaluated after a completed thermal cycles. Nevertheless, since particle protrusion is main thermal cycling result concluded in AZ31/SiC, it can be inferred that frictional sliding along interface and interfacial debonding is predominant cause pertaining to thermal cycling damage. Thus, the presence of some microcracking, microvoids, and the weak interfacial bond, should possibly facilitate the onset of frictional sliding when thermal cycling occurs.

In contrast to many other composites studied previously, e.g. Al/C composites^60^, AZ31/SiC does not observe a gradual accumulation of damage, with negligible degradation in property, occurring at the beginning of the cycling process. After initial interfacial debonding, the generation of stresses may not be as very profound, and further damage accumulation (coalescence of existing flaws) will be slower. It should be noted that composites with strong interfaces have proved to be more resistant to mechanical property devaluation in a relatively transient thermal environment^70^. Therefore, the formation of a stronger interfacial bond through the formation of carbide at the interface probably improves the mechanical response of AZ31/SiC when exposed to cyclic thermal conditions^66^.

### Mechanism

**Figure 19:**
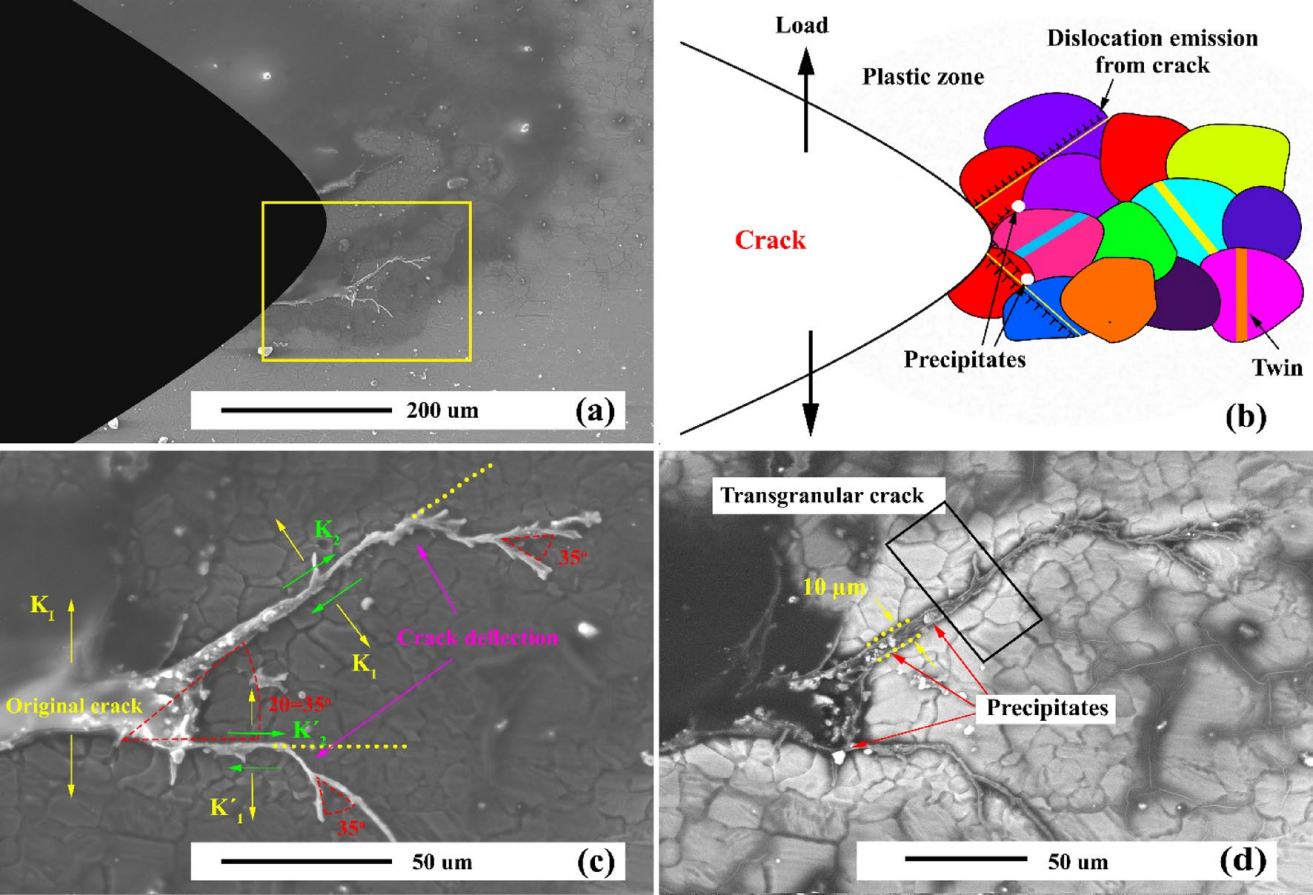
(**a**) SEM micrograph of aged unreinforced alloy after thermal cycles at 350 °C, and (**b**) schematics of crack propagation through the grains, (**c**) higher magnification of micrograph (**a**) in SE mode, and (**b**) higher magnification of micrograph (**a**) in BSE mode.

Figure 19 represents the thermal fatigue cracking micrographs of aged unreinforced alloy samples. Apparently, in the first stage, thermal fatigue crack grown transgranular, which was in the direction to groove starting from the notch's tip. CL grew with an increase in the number of cycles. The crack width of various samples declined; however, its thickness did not exceed 10 µm. This is while the crack width has decreased with reaching the precipitates, followed by crack deflection and bifurcation, indicating crack growth resistance mechanisms. Cracks in the as-cast sample grew straight (Fig. 18), while in the aged sample, they grew as branches (Fig. 19a).

Obviously, under conditions similar to those used in these experiments, more than 900 thermal cycles are usually required to cause any measurable damage to the samples. Hence, recorded CTE in these experiments is completely irrelevant to the occurrence of matrix microstructural changes by plastic deformation. Therefore composite was prepared through stir casting method at elevated temperature. It consists of residual thermal stresses at RT because of a mismatch in coefficients on matrix/reinforcement thermal expansion^71^; the magnitude of these stresses is the minimum stress required for the creep to occur in the matrix. It seems that the matrix in AZ31/SiC composite bears tensile stresses while reinforcements particles bear compressive stresses. Since composites samples during heating, internal thermal tensile stress in matrix drops to a minimum, and as it is heated further, there may be a compressive stresses generation.

On the contrary, when composites are cooled, internal thermal stresses behave oppositely. Indeed, it is assumed that internal stresses may be concentrated near the interface of matrix/reinforcement and particularly at the sharp side of reinforcement. Also, internal thermal stresses could go beyond the yield stress of the matrix alloy at the evaluated temperature. Then relaxation will occur by new dislocations generation and matrix plastic deformation. Plastic deformation could appear as a grain boundary sliding, twinning, or dislocation glide at higher temperatures depending on the crystallographic matrix structure. Compressive deformation, which appears during the heating process, is expected to lead to some form of diffusional creep. In contrast, plastic deformation appearing on cooling can cause twinning and dislocation glide. Therefore, in support of experimental observations, a larger, more considerable thermal mismatch is expected to occur during cooling at lower temperatures. As suggested earlier, damage of internal thermal stresses reproduced in the thermal cycling can possibly be correlated^71–73^.

In Fig. 19d, the crack encountered a group of precipitates with two paths for propagation; it grew along with the particle interface. During the entire growth process, the crack may affect particles and move along with less strong positions, namely cavities and weak interfaces. At the crack tip, the coefficient of stress intensity is associated with bond strength concerning the interface of reinforcement/matrix. There is a considerable distinction between CTE of constituents. Thus, during cycles, thermal stresses arise from interfaces. Thermal stress caused by thermal fatigue may weaken the properties of composite material. However, propagation occurs in the matrix when the alloy matrix’s yield strength is less than the strength of the interface and when the crack path does not collide with any interfaces. During the entire growth process, a crack may affect particles and move along with less strong positions, namely cavities and weak interfaces. At the crack tip, the coefficient of stress intensity is associated with bond strength concerning the interface of reinforcement/matrix. There is a considerable distinction between CTE of constituents. Thus, during cycles, thermal stresses arise from interfaces. Thermal stress caused by thermal fatigue may weaken the properties of composite material. However, propagation occurs in a matrix in the case in alloy matrix yield strength is less than the strength of interface and when the crack path does not collide with any interfaces.

**Figure 20:**
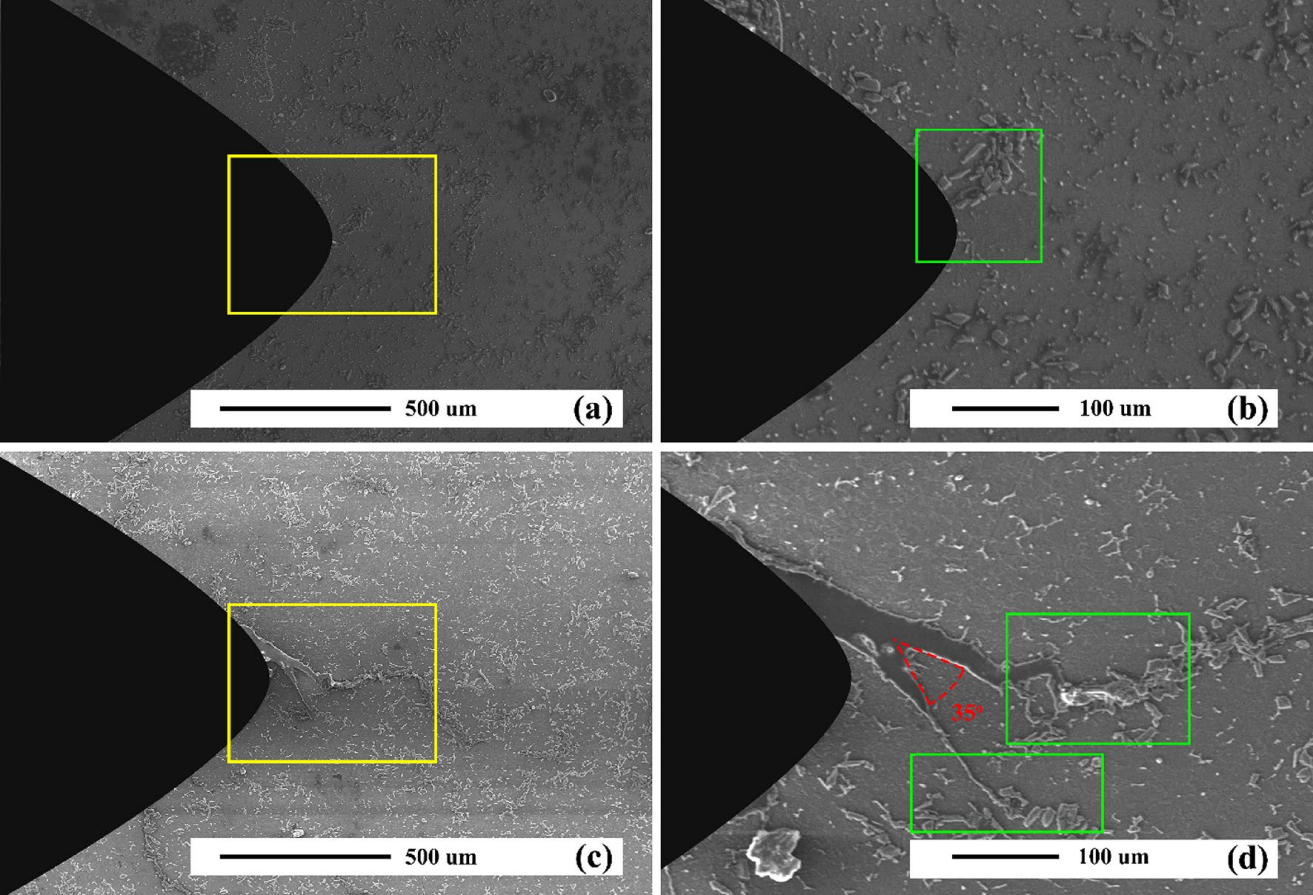
SEM micrograph of as-cast AZ31/5%SiC after thermal cycles at 350 °C (**a**) and (**b**) 900 thermal cycles, and (**c**) and (**d**) 900 thermal cycles.

Figure 20 reveals the SEM micrographs of an AZ31/5%SiC composite sample after 300 and 900 thermal cycles. As can be seen, the sample under 300 thermal cycles has no crack, which in the micrograph with higher magnification can be seen in the presence of a cluster of reinforcing particles in the notch tip, which itself can prevent the nucleation of the crack. In the sample, under 900 thermal cycles, the reinforcement particles have prevented the straight growth of cracks in the composite matrix. Also, crack width has decreased sharply after colliding with reinforcement particles, indicating that these particles have enhanced the thermal fatigue resistance of the composite. Since their distribution and thermal load may alter the propagation of thermal fatigue cracks, crack growth can be inhibited by particles. Although the crack growth process encounters many particles as barriers, the crack grows rapidly after a definite interval on cycles. After that, it crosses the next barrier. Its growth, as a whole, must bypass a particle that has a longer path and it needs more energy to travel the same path. Reinforcement particles and participants control crack growth. Straight growth of crack is usually prevented in as-cast and aged samples by SiC particles and precipitates.

**Figure 21:**
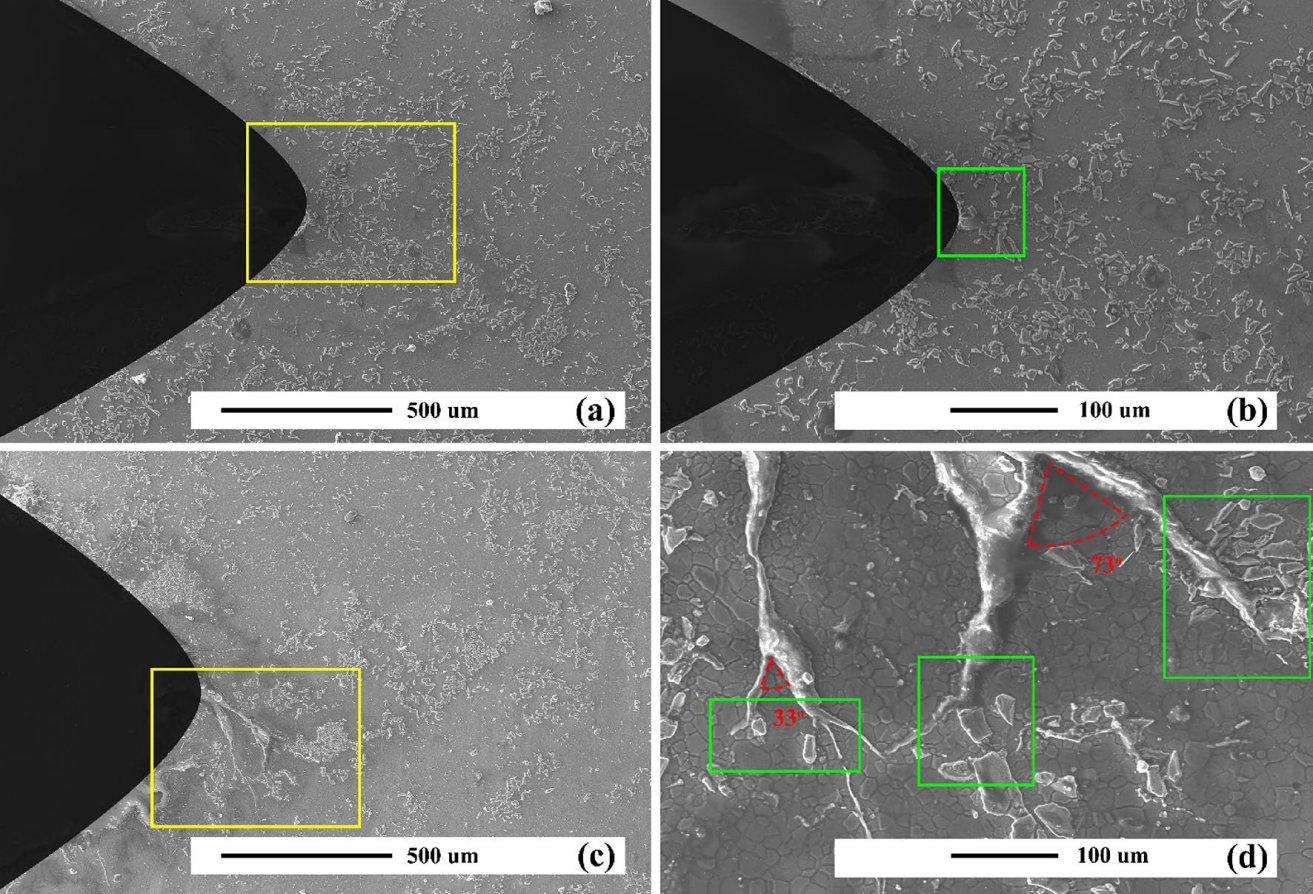
SEM micrograph of as-cast AZ31/10%SiC after thermal cycles at 350 °C (**a**) and (**b**) 300 thermal cycles, and (**c**) and (**d**) 900 thermal cycles.

It is well-known that SiC particles as reinforcement materials are critical factors in the crack process in which cracks should pass them. Figure 21 presents AZ31/SiC composites in which cracks started to grow and propagated slowly and quickly. But, after a while, the width of the crack rose at the notch of the sample, and it then facilitated its growth. After heating and cooling cycles, the interface of particle and matrix often has a relatively low strength. This is because strength at interfaces is much less than thermal stresses. Combined with defects, this stress makes cracks initiate and propagate. Moreover, a crack may grow in the matrix if the matrix has lower values of strength compared to that of the interface, or the crack does not face any interfaces.

**Figure 22:**
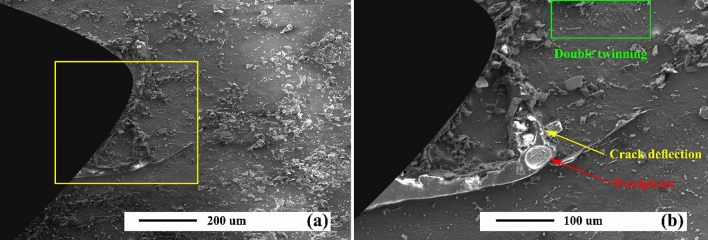
(**a**) SEM micrograph of aged AZ31/10%SiC after thermal cycles at 150 °C, and (**b**) higher magnification.

Figure 22 reveals the crack propagation in the aged AZ31/10%SiC composite sample. As can be seen, the straight crack was deflected by a precipitate and further stopped by the reinforcing particles. The growth process of thermal fatigue crack, as a whole, has two stages, including initiation and propagation. During this process, the length and width of cracks grow similarly in the sense that crack propagation advances consistently at different stages. Moreover, the width, and length of a crack caused by thermal fatigue in the propagation process experience an increase and a decrease. Although CL concerning two side faces of unreinforced alloy remains the same, it is different in composites, particularly change in width is opposite. It should be mentioned that observed trends are as follows. First, the platform period is where CL remains almost stable under several heating and cooling cycles. Then, as CL grows sharply, the crack enters fast to the next step, which is considered the jumping period. As steps increases, thermal fatigue cracks are likely to grow slowly and then quickly in a cyclic way till their speed is stabilized. Although crack growth encountered many obstacles (reinforcements and precipitates), the crack propagates quickly in the initial duration of both types of cycles and then may face a new hurdle. In other words, it is notable that propagation may advance slowly and quickly. However, there was a random distribution of the path of fatigue crack in composite reinforced by particles. Indeed, propagation may be hindered, and particles will change the growth path.

Nevertheless, it should be noted that during the process of crack growth, the existence of individual particles cannot be seen. Uniform distribution on a group of particles is a critical factor in impeding the growth of the main crack. Composite samples do not represent any turning points where initiation changed to propagation. As shown in Fig. 22, this point is where CL and cycles are 200 µm and 900, respectively.

**Figure 23:**
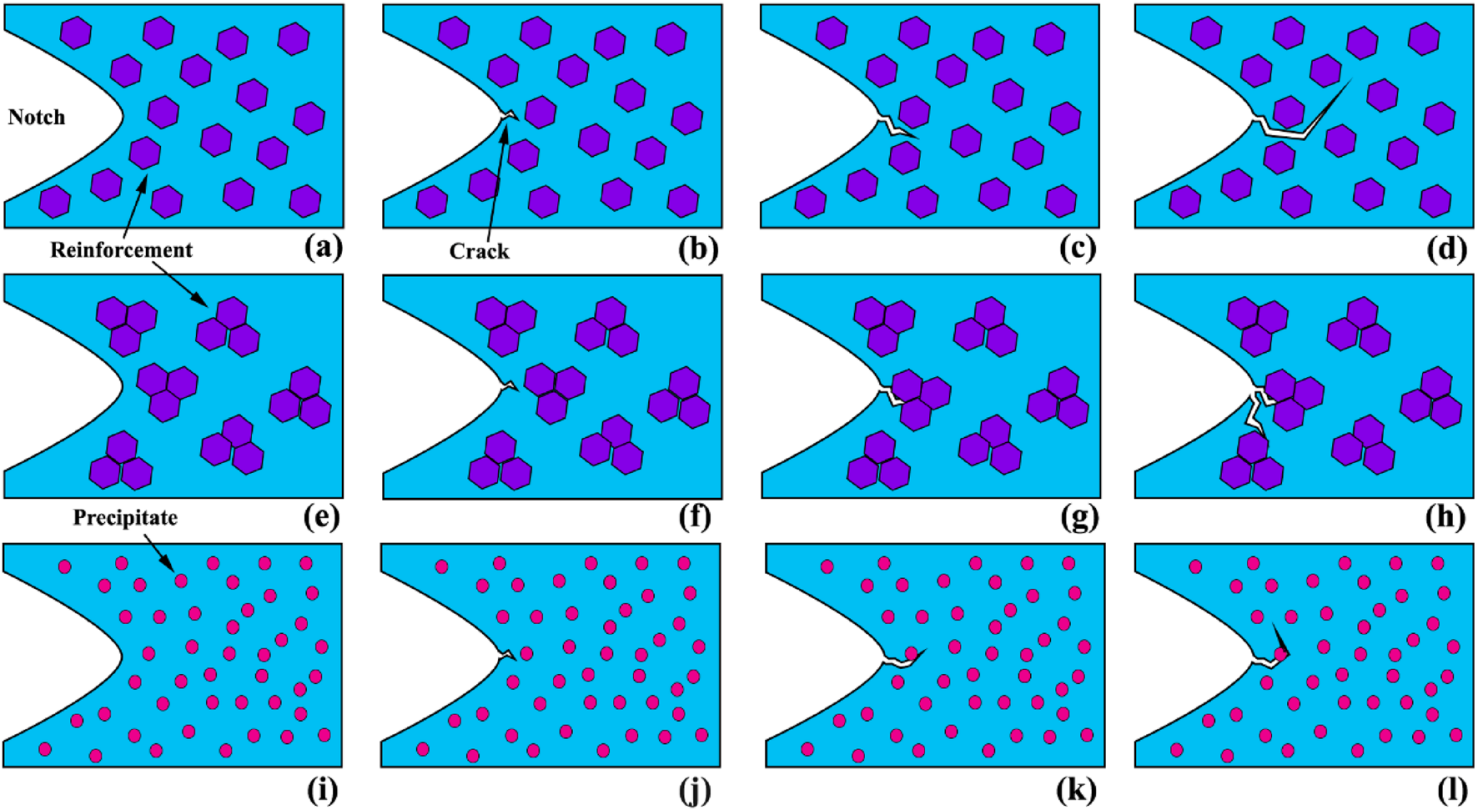
Schematics of crack propagation resistance mechanism during thermal cycles in composite and precipitated hardened sample: (**a**–**d**) composite with uniform reinforcement distributions, (**e**–**h**) composite with cluster reinforcement distributions, and (**i**–**l**) aged sample.

The thermal-fatigue crack growth model in AZ31/SiC composite is demonstrated in Fig. 23. The horizontal and vertical surfaces of each stage are attributed to the slow and rapid propagation of thermal fatigue cracking. Based on the figure in low cycles, crack growth is slow, which attributes to activation energies for crack nucleation (Fig. 23a,b). As cycles increase, CL reaches equilibrium, indicating that the crack growth rate gradually becomes stable, followed by an increase in thermal fatigue crack growth rate (Fig. 23c). Applying various cycles does not alter LC, indicating a stage that can be called starting period. After that, when CL increases sharply, the crack goes to the next stage, called the jump period. Hence, length varies modestly. Then it increases significantly. In other words, they may change slowly or quickly before growth rates on cracks' fast and slow growth become similar. In this stage in composite and aged samples, reinforcement/precipitates act as a deflection agent. Based on Fig. 23 three mechanisms can be activated for crack resistance. First, in the relatively homogeneous composite sample, the elastic field around the reinforcement particle acts as a crack deflection agent, which causes increasing CL and decreasing thermal load (Fig. 23d). Second, in an inhomogeneous composite sample, the cluster of reinforcement acts as a barrier to crack growth. Furthermore, due to the high strength of ceramic particles, the crack cannot fracture the particle and also cannot be deflected due to a cluster of reinforcement, which causes new crack nucleated up to reach the cluster of particles (Fig. 23e–h). Third, in the percent of the precipitates, crack growth in the path of the precipitates cause an increasing curvature radius of the crack tip and further cause crack deflection (Fig. 23i–l).

### Crack growth model

Figure 19 illustrates the calculated routes of a subsection concerning crack produced by 900 cycles. As expected, the longer one arrests the short one and proceeds, the crack returns to its original direction of propagation. It should be noted that the overload-induced bifurcation phenomenon brings the about a great size of zones with nonelastic deformation wherein branches develop. Ithe critical effect of the mentioned areas is that they may close cracks, especially when merged into effect of the bifurcation phenomenon. Nevertheless, in the experiments, thermal cycles have been controlled not to have a closing effect, thus eliminating nonelastic regions around the crack tip. Far from this mentioned area, an observed zigzag pattern often arises from microstructural variation; nevertheless, kinks under 35° never impact stress intensity factors, as illustrated in Fig. 19. However, a final path of crack propagation caused by bifurcated often appears in a trans-granular shape.

Many factors, such as multi-axial stresses, overloads, and microstructural inhomogeneities often force fatigue cracks to digress from the growth direction of Mode I^74^. Figure 19 displays that this deviation may generate kinks or branches produced in a crack. In addition, a fatigue crack that deviated from Mode I plane is likely to induce conditions with mixed-mode around the crack edge when Mode I is prevalent in far-field stress. Figure 19 shows that this mode of stress adjacent to a long subdivision of cracks produces both modes of stress intensity, but it yields k_1_ and k_2_ adjacent to a smaller branch. Considering small factors of divided ones compared to that of a straight one with similar lengths, branching may postpone its next growth.

Furthermore, roughness observed on fracture surfaces that arises from such branching may change the level at which the crack is closed. Then, perturbations can be developed as the crack grows^75–77^. According to the results, minimal differences between both CLs branches (Fig. 19c,d) are large and can produce shorter branches to be arrested while longer subdivisions can propagate. Accordingly, when the rate and direction of cracks are satisfied, the curve of subdivisions. Consequently, it is believed that the fastest branch proceeds growing whereas many advance around the entire path. On the other hand, all others are hindered owing to the effect of shielding. The mentioned feature also has widely detected engineering parts, specifically on an airplane wheel rim^78–80^.

Three techniques can be applied to determine factors of stress intensity (FSI) around curved paths of cracks. First, the technique of displacement correlation; second, a technique that computes the release rate of potential energy by using an integral method on a modified crack-closure; third, equivalent domain integral (EDI), which computes J-integral^78^. Both Modes of I and II and FSI K_I_ and K_II_ were utilized to calculate an equivalent FSI K_eq_. The growth rate of the thermal fatigue crack could then be determined by using the range D_Keq_ on stress intensity by a McEvily model^81^:

2 $$ \frac{da}{{dN}} = A \times \left( {\Delta K_{eq} - \Delta K_{th} } \right)^{m} $$

3 $$ \Delta c = \Delta b \times \left( {\frac{{\Delta K_{c} - \Delta K_{th} }}{{\Delta K_{b} - \Delta K_{th} }}} \right)^{m} $$

where the Modes I and II FSI are evaluated for CLs with a small bifurcation of branch lengths b_0_ and c_0_, which b_0_ > c_0_ and with angle 2θ (marked Fig. 19c).

Two modes on subdivisions were achieved with angles of 35° < 2θ < 73° about bifurcation. It should be noted that as cracks growth initiates in weak material and then propagates into strong one, the thermal load induced bifurcated cracks may have initial branch lengths in a range of 10–100 µm, with 2θ = 35°, in the unreinforced alloy, and 73°, in composite^82^.

Bifurcation angle 2θ is a vital parameter wherein k_2_ vanishes on uniformly deviated crack because the other deviation may propagate. On the other hand, k_2_ is equal to zero, which means a route's deviation may occur. Therefore, both branches may grow at an angle θ = 35° concerning the horizontal direction. Then, using Eq. (4) ^78^, the k_1_ and k_2_ were utilized in calculations of equivalent FSI K_b_ concerning those subdivisions. They can then detect growth features when bifurcation occurs.

4 $$ K_{eq} = \frac{1}{4}\left( {3\cos \frac{\theta }{2} + \cos \frac{3\theta }{2}} \right) \times K_{I} - \frac{3}{4}\left( {\sin \frac{\theta }{2} + \sin \frac{3\theta }{2}} \right) \times K_{II} $$

Figure 19 shows constancy in K_b_/K_I_ ratio, about 0.81, for regularly bifurcated cracks when angles are 2θ < 35°. However, at the time of computation regarding stress intensity factor these cracks, special care must be taken. Effective FSI increases considerably when propagation initiates. For example, regarding a regularly bifurcated crack when angles of branches are 2θ = 73°, K_b_/K_I_ is around 0.77, but it propagates modestly with less than 0.1 b_0_, it rises rapidly to 0.751. Therefore, the reduction of K_b_/K_I_ for 2θ = 35°, as presented in Fig. 21 may be correct when propagation initiates, which then immediately increases to approximately 0.8. Moreover, the initial propagation longer branch direction is lower 35°, independent of considered bifurcation angle 2θ, as shown in Fig. 19. Therefore, for values of 2θ < 73°, a deflection can be detected when propagation begins. This result has also been reported by Lankford^74^, who performed several tests concerning overload fatigue crack on alloy using SEM micrographs and reported that growth postpone arises from the bifurcation. Reportedly, it probably grows shortly parallel to overload-induced bifurcation before a rapid branching within its route occurs, as can be observed in Fig. 21. The mentioned branching results in a sharp rise in Mode I of stress intensity factor instantly after the start of propagation leading to a lower postponed effect according to the bifurcation without the propagation phase. Nonetheless, when equivalent stress intensity spans of both subdivisions are less than D_Kth_, then the main crack will be arrested, and thus no rapid branching may be developed.

Figure 21 illustrates defined routes on deviated crack produced as CL was a = 200 µm. Also, the primary one faced kinking before the bifurcation phenomenon. The zigzag pattern is evident at its propagation; nevertheless, it does notimpact stress intensity factor data. SEM micrographs also demonstrates through-the-thickness condition concerning bifurcation front in all samples.

Figure 21 reveals open crack measurements in the sample after 300 and 900 thermal cycles, which caused bifurcation. This is because the initial load usually stayed less than the lowest value in the range of imposed loads. Thus, we can infer that crack closure cannot explain the measured effect of retardation. Indeed, the bifurcation phenomenon declined the level of closure by 25% because of increased compliance caused by crack branches. The effect of retardation, which may be attributed to a drop in Kop, may be inconsistent with neither of postpone postponing techniques based on the crack's closure. Then, it can be suggested that bifurcation is the primary postponement mechanism. The advantages of the growth path on a crack caused by thermal fatigue outweigh its disadvantages by improving its resistance. Figure 21 reveals shown concerning crack caused by thermal fatigue in an unreinforced sample. As illustrated in figure, each period comprises many rough microfracture surfaces. This proves hurdles against the growth of cracks. Accordingly, a crack can grow further or combine with tiny ones after overcoming various obstacles. Eventually, it mainly grew along the matrix/particle interface^39,83–85^.

## Conclusions

This research study discussed the behavior of cracks produced by thermal fatigue tested with a V-shape notch sample in Mg/SiC composites, consisting of 5 and 10wt% SiC particles, and particularly in AZ31 alloy, which were fabricated by stir casting, at various of cycles of heating and cooling at 150 and 350 °C. Moreover, the crack growth and its mechanism and growth rule, as well as its morphology, were examined. The results are summarized below:

1. Cracks initiate during the first thermal cycles and when their length reach 200 µm, they start to propagate in the matrix. This stage is not only the main stage but also may propagate slowly and quickly during the process.
2. Based upon the OM and SEM results, the trans-granular crack propagated in the unreinforced alloy. After that, it entered into the matrix/reinforcement and matrix/precipitates interfaces which deflect the crack propagations. During thermal fatigue, with an increase in the number of cycles, a crack growth barrier or postpone arises from the bifurcation. Then microcracks emerged.
3. The second stage of growth concerning cracks was propagation which first grew slowly. However, after a while, when it reached a definite number of cycles, it grew faster. This difference was observed on the fracture surface with the shape of steps representing small jagged crack paths.
4. The primary resistance mechanism of crack growth in aged and reinforced Mg alloys is the deflection by precipitates and barriers by a cluster of reinforcement. Furthermore, crack deflection due to the elastic field of the matrix around the reinforcement particle postponed the crack growth.

## Data Availability

All data generated or analyzed during this study are included in this published article.

## References

[1] Moheimani SK, Azadeh K, Khademzadeh S, Tayebi M, Rajaee A, Saboori A (2022). Tribological behaviour of AZ31 magnesium alloy reinforced by bimodal size B4C after precipitation hardening. J. Magnes. Alloy..

[2] Tayebi M, Nategh S, Najafi H, Khodabandeh A (2020). Tensile properties and microstructure of ZK60/SiCw composite after extrusion and aging. J. Alloys Compd..

[3] Tayebi M, Bizari D, Hassanzade Z (2020). Investigation of mechanical properties and biocorrosion behavior of in situ and ex situ Mg composite for orthopedic implants. Mater. Sci. Eng. C..

[4] Tayebi M, Najafi H, Nategh S, Khodabandeh A (2021). Creep behavior of ZK60 Alloy and ZK60/SiCw composite after extrusion and precipitation hardening. Met. Mater. Int..

[5] Bigdeli F, Javidi M, Pakshir M, Khezrloo A, Tayebi M (2021). Risk assessment of the corrosion resistance performances for epoxy coatings under drilling environments using AHP method. Int. J. Press. Vessel. Pip..

[6] Wang X, Yang J, Chi P, Bahonar E, Tayebi M (2021). Effects of the microstructure and precipitation hardening on the thermal expansion behavior of ZK60 magnesium alloy. J. Alloys Compd..

[7] Lv B-J, Wang S, Xu T-W, Guo F (2021). Effects of minor Nd and Er additions on the precipitation evolution and dynamic recrystallization behavior of Mg–6.0Zn–0.5Mn alloy. J. Magnes. Alloy..

[8] Ye HZ, Liu XY (2004). Review of recent studies in magnesium matrix composites. J. Mater. Sci..

[9] Long F, Chen G, Zhou M, Shi Q, Liu Q (2021). Simultaneous enhancement of mechanical properties and corrosion resistance of as-cast Mg-5Zn via microstructural modification by friction stir processing. J. Magnes. Alloy..

[10] Li H, Wang K, Xu G, Jiang H, Wang Q, Wang Y (2021). Effective inhibition of anomalous grain coarsening in cast AZ91 alloys during fast cooling via nanoparticle addition. J. Magnes. Alloy..

[11] Li J, Dong Z, Yi X, Wu D, Chen R (2021). Twin evolution in cast Mg-Gd-Y alloys and its dependence on aging heat treatment. J. Magnes. Alloy..

[12] Kumar KCK, Kumar BR, Rao NM (2021). Microstructural, mechanical characterization, and fractography of AZ31/SiC reinforced composites by stir casting method. Silicon.

[13] Zhu Y-X, Song G-L, Wu P-P (2021). Self-repairing functionality and corrosion resistance of in-situ Mg-Al LDH film on Al-alloyed AZ31 surface. J. Magnes. Alloy..

[14] Fajardo S, Miguélez L, Arenas MA, de Damborenea J, Llorente I, Feliu S (2021). Corrosion resistance of pulsed laser modified AZ31 Mg alloy surfaces. J. Magnes. Alloy..

[15] Li J, Qiu Y, Yang J, Sheng Y, Yi Y, Zeng X, Chen L, Yin F, Su J, Zhang T, Tong X, Guo B (2021). Effect of grain refinement induced by wire and arc additive manufacture (WAAM) on the corrosion behaviors of AZ31 magnesium alloy in NaCl solution. J. Magnes. Alloy..

[16] Vaidya AR, Lewandowski JJ (1996). Effects of SiCp size and volume fraction on the high cycle fatigue behavior of AZ91D magnesium alloy composites. Mater. Sci. Eng. A..

[17] Zhao M, Zheng MY, Wu K, Peng WF, Lei TC (2003). Effect of thermal cycling on the mechanical properties of SiCw/ZK60 magnesium matrix composite. J. Mater. Sci. Lett..

[18] Weiler JP (2021). Exploring the concept of castability in magnesium die-casting alloys. J. Magnes. Alloy..

[19] Badini C, Fino P, Musso M, Dinardo P (2000). Thermal fatigue behaviour of a 2014/Al2O3-SiO2 (Saffil®fibers) composite processed by squeeze casting. Mater. Chem. Phys..

[20] Guan K, Egusa D, Abe E, Zhang J, Qiu X, Yang Q, Meng J (2021). Microstructures and mechanical properties of as-cast Mg-Sm-Zn-Zr alloys with varying Gd contents. J. Magnes. Alloy..

[21] Tong X, Wu G, Zhang L, Wang Y, Liu W, Ding W (2020). Microstructure and mechanical properties of repair welds of low-pressure sand-cast Mg–Y–RE–Zr alloy by tungsten inert gas welding. J. Magnes. Alloy..

[22] Wang T, Whalen S, Ma X, Silverstein J, Das H, Pallaka MR, Ortiz A, Roosendaal T, Upadhyay P, Kappagantula KS (2021). Friction-based riveting technique for AZ31 magnesium alloy. J. Magnes. Alloy..

[23] Song B, Wang M, Shi R, Du Z, Guo N, Wang F, Guo S (2021). Promoting hybrid twins structure to reduce yield asymmetry of rolled AZ31 plates by combining side-rolling and torsion. J. Magnes. Alloy..

[24] Chelliah NM, Singh H, Surappa MK (2016). Correlation between microstructure and wear behavior of AZX915 Mg-alloy reinforced with 12 wt% TiC particles by stir-casting process. J. Magnes. Alloy..

[25] Aravindan S, Rao PV, Ponappa K (2015). Evaluation of physical and mechanical properties of AZ91D/SiC composites by two step stir casting process. J. Magnes. Alloy..

[26] Rashad M, Pan F, Liu Y, Chen X, Lin H, Pan R, Asif M, She J (2016). High temperature formability of graphene nanoplatelets-AZ31 composites fabricated by stir-casting method. J. Magnes. Alloy..

[27] D SK, KNS S, SC T, K R, Poddar P, SB VS (2017). Microstructure, mechanical response and fractography of AZ91E/Al2O3 (p) nano composite fabricated by semi solid stir casting method. J. Magnes. Alloy..

[28] Kumar A, Kumar S, Mukhopadhyay NK (2018). Introduction to magnesium alloy processing technology and development of low-cost stir casting process for magnesium alloy and its composites. J. Magnes. Alloy..

[29] Maurya M, Kumar S, Bajpai V (2018). Assessment of the mechanical properties of aluminium metal matrix composite: A review. J. Reinf. Plast. Compos..

[30] Dwivedi SP, Maurya M, Saxena A, Sharma S (2021). Synthesis and characterization of spent alumina catalyst and grinding sludge reinforced aluminium-based composite material. Proc. Inst. Mech. Eng. Part C J. Mech. Eng. Sci..

[31] Maurya M, Kumar S, Bajpai V, Maurya NK (2020). Process parameters, development and applications of stir cast composite: A review. Mater. Test..

[32] Maurya NK, Maurya M, Srivastava AK, Dwivedi SP, Kumar A, Chauhan S (2020). Investigation of mechanical properties of Al 6061/SiC composite prepared through stir casting technique. Mater. Today Proc..

[33] Zhao L, Xin Y, Jin Z, Wang J, Feng B, Liu Q (2019). Thermal stability of different texture components in extruded Mg–3Al–1Zn alloy. J. Magnes. Alloy..

[34] Kang S-H, Han D-W, Kim H-K (2020). Fatigue strength evaluation of self-piercing riveted joints of AZ31 Mg alloy and cold-rolled steel sheets. J. Magnes. Alloy..

[35] Wang X, He C, Li X, Li L, Liu Y, Wang Q (2021). Effect of long-period stacking ordered structure on very high cycle fatigue properties of Mg-Gd-Y-Zn-Zr alloys. J. Magnes. Alloy..

[36] Bazhenov VE, Koltygin AV, Sung MC, Park SH, Tselovalnik YV, Stepashkin AA, Rizhsky AA, Belov MV, Belov VD, Malyutin KV (2021). Development of Mg–Zn–Y–Zr casting magnesium alloy with high thermal conductivity. J. Magnes. Alloy..

[37] Ning J, Na S-J, Zhang L-J, Wang X, Long J, Cho W-I (2021). Improving thermal efficiency and stability of laser welding process for magnesium alloy by combining power modulation and subatmospheric pressure environment. J. Magnes. Alloy..

[38] Li S, Yang X, Hou J, Du W (2020). A review on thermal conductivity of magnesium and its alloys. J. Magnes. Alloy..

[39] Pan L, Han J, Yang Z, Li X, Wang J, Li Z, Li W (2017). Thermal fatigue crack behavior of SiCp/A356 composites prepared by stirring casting. Results Phys..

[40] Q. Xin, Durability and reliability in diesel engine system design, in: 2013: pp. 113–202. 10.1533/9780857090836.1.113.

[41] Huang Y, Hort N, Dieringa H, Maier P, Kainer K (2006). Investigations on thermal fatigue of aluminum- and magnesium-alloy based composites. Int. J. Fatigue Int. J. Fatigue..

[42] Rohatgi PK, Gupta N, Alaraj S (2005). Thermal expansion of aluminum-fly ash cenosphere composites synthesized by pressure infiltration technique. J. Compos. Mater..

[43] Birol Y (2010). Thermal fatigue testing of Inconel 617 and Stellite 6 alloys as potential tooling materials for thixoforming of steels. Mater. Sci. Eng. A..

[44] Badini C, Fino P, Musso M, Dinardo P, Birol Y, Huang Y, Hort N, Dieringa H, Maier P, Kainer K, Kumar KCK, Kumar BR, Rao NM, Rohatgi PK, Gupta N, Alaraj S, Vaidya AR, Lewandowski JJ, Wu CM, Han GW, Ye HZ, Liu XY, Zhao M, Zheng MY, Wu K, Peng WF, Lei TC (2006). Thermal fatigue behaviour of SiCp/Al composite synthesized by metal infiltration. Compos. Part A Appl. Sci. Manuf..

[45] Zare R, Sharifi H, Saeri MR, Tayebi M (2019). Investigating the effect of SiC particles on the physical and thermal properties of Al6061/SiCp composite. J. Alloys Compd..

[46] Tayebi M, Jozdani M, Mirhadi M (2019). Thermal expansion behavior of Al–B4C composites by powder metallurgy. J. Alloys Compd..

[47] Tayebi M, Tayebi M, Rajaee M, Ghafarnia V, Rizi AM (2021). Improvement of thermal properties of Al/Cu/SiC composites by tailoring the reinforcement microstructure and comparison to thermoelastic models. J. Alloys Compd..

[48] Kumar S, Dieringa H, Kainer K-U (2005). Effect of particulate content on the thermal cycling behaviour of the magnesium alloy based hybrid composites. Compos. Part A Appl. Sci. Manuf..

[49] Kerenciler H, Gündüz S, Erden MA, Türkmen M, Karabulut H (2016). Effect of aging on the microstructure and mechanical properties of magnesium alloy AZ31. Met. Sci. Heat Treat..

[50] Sharifi H, Eidivandi V, Tayebi M, Khezrloo A, Aghaie E (2017). Effect of SiC particles on thermal conductivity of Al-4%Cu/SiC composites. Heat Mass Transf. Und Stoffuebertragung..

[51] Yang Q, Dai Q, Lou C, Dai J, Zhang J, Jiang B, Pan F (2019). Twinning, grain orientation, and texture variations in Mg alloy processed by pre-rolling. Prog. Nat. Sci. Mater. Int..

[52] Li WX, Nie YF, Wang DD (2011). Mechanical behavior of CNTs/SiCp/AZ91D magnesium matrix composites. Mater. Sci. Forum..

[53] Zhang Z, Yang F, Zhang H, Zhang T, Wang H, Xu Y, Ma Q (2021). Influence of CeO2 addition on forming quality and microstructure of TiCx-reinforced CrTi4-based laser cladding composite coating. Mater. Charact..

[54] Li X, Yang X, Yi D, Liu B, Zhu J, Li J, Gao C, Wang L (2021). Effects of NbC content on microstructural evolution and mechanical properties of laser cladded Fe50Mn30Co10Cr10-xNbC composite coatings. Intermetallics.

[55] Saleh B, Jiang J, Fathi R, Xu Q, Wang L, Ma A (2020). Study of the microstructure and mechanical characteristics of AZ91–SiCp composites fabricated by stir casting. Arch. Civ. Mech. Eng..

[56] Lu L, Lim CYH, Yeong WM (2004). Effect of reinforcements on strength of Mg9%Al composites. Compos. Struct..

[57] Kumar S, Ingole S, Dieringa H, Kainer K-U (2003). Analysis of thermal cycling curves of short fibre reinforced Mg-MMCs. Compos. Sci. Technol..

[58] Kumar S, Mondal AK, Dieringa H, Kainer K-U (2004). Analysing hysteresis and residual strains in thermal cycling curves of short fibre reinforced Mg-MMCs. Compos. Sci. Technol..

[59] E. Carreno-Morelli, E. Urreta, R. Schaller, International conference on fatigue of composites, Societe Francaise de Metallurgie et de Materiaux, in: Paris, 1977: p. 112.

[60] M.M. Avedesian, H. Baker, A.S.M.I.H. Committee, *ASM Specialty Handbook: Magnesium and Magnesium Alloys*, ASM International, 1999. https://books.google.ca/books?id=0wFMfJg57YMC.

[61] Li S, Sun L, Sun Z, Wang Z (1991). Thermal residual stress relaxation at surface of SiC/Al composite. Scr. Metall. Mater..

[62] Wang H, Xie J, Chen Y, Liu W, Zhong W (2022). Effect of CoCrFeNiMn high entropy alloy interlayer on microstructure and mechanical properties of laser-welded NiTi/304 SS joint. J. Mater. Res. Technol..

[63] Zhong Y, Xie J, Chen Y, Yin L, He P, Lu W (2022). Microstructure and mechanical properties of micro laser welding NiTiNb/Ti6Al4V dissimilar alloys lap joints with nickel interlayer. Mater. Lett..

[64] Rudajevová A, Balik J, Lukáč P (2000). Thermal properties of a magnesium hybrid composite: QE22 alloy reinforced with 20 Vol% SiC particles and 5 Vol% Al2O3 fibres. Sci. Eng. Compos. Mater..

[65] Bayani H, Saebnoori E (2009). Effect of rare earth elements addition on thermal fatigue behaviors of AZ91 magnesium alloy. J. Rare Earths..

[66] Russell-Stevens M, Todd R, Papakyriacou M (2005). The effect of thermal cycling on the properties of a carbon fibre reinforced magnesium composite. Mater. Sci. Eng. A..

[67] Buffière J-Y, Savelli S, Jouneau PH, Maire E, Fougères R (2001). Experimental study of porosity and its relation to fatigue mechanisms of model Al–Si7–Mg0.3 cast Al alloys. Mater. Sci. Eng. A..

[68] Viala J, Claveyrolas G, Bosselet F, Bouix J (2000). The chemical behavior of carbon fibres in magnesium base Mg-Al alloys. J. Mater. Sci..

[69] Yoda S, Takahashi R, Wakashima K, Umekawa S (1979). Fiber/matrix interface porosity formation in tungsten fiber/copper composites on thermal cycling. Metall. Mater. Trans. A..

[70] Kyono T, Kuroda E, Kitamura A, Mori T, Taya M (1988). Effects of thermal cycling on properties of carbon fiber/aluminum composites. J. Eng. Mater. Technol..

[71] Chmelík F, Lukáč P, Kiehn J, Mordike BL, Kainer K-U, Langdon TG, Chmelík F, Lukac P, Kiehn J, Mordike BL, Kainer K-U, Langdon TG (2002). Characteristics of thermal cycling in a magnesium alloy composite. Mater. Sci. Eng. A..

[72] Carreno-Morelli E, Urreta S, Schaller R (2000). Mechanical spectroscopy of thermal stress relaxation at metal–ceramic interfaces in Aluminium-based composites. Acta Mater..

[73] Wang Z, Qiang H, Wang J, Duan L (2022). Experimental investigation on fracture properties of HTPB propellant with circumferentially notched cylinder sample, propellants. Explos. Pyrotech..

[74] Lankford J, Davidson D (1981). The effect of overloads upon fatigue crack tip opening displacement and crack tip opening/closing loads in aluminum alloys. Adv. Fract. Res..

[75] Suresh S (1983). Crack deflection: Implications for the growth of long and short fatigue cracks. Metall. Trans. A..

[76] Bai Y, Nardi DC, Zhou X, Picón RA, Flórez-López J (2021). A new comprehensive model of damage for flexural subassemblies prone to fatigue. Comput. Struct..

[77] Yang M, Li C, Said Z, Zhang Y, Li R, Debnath S, Ali HM, Gao T, Long Y (2021). Semiempirical heat flux model of hard-brittle bone material in ductile microgrinding. J. Manuf. Process..

[78] Meggiolaro MA, Miranda ACO, Castro JTP, Martha LF (2005). Stress intensity factor equations for branched crack growth. Eng. Fract. Mech..

[79] Zhou X, Bai Y, Nardi DC, Wang Y, Wang Y, Liu Z, Picón RA, Flórez-López J (2022). Damage evolution modeling for steel structures subjected to combined high cycle fatigue and high-intensity dynamic loadings. Int. J. Struct. Stab. Dyn..

[80] Liang L, Xu M, Chen Y, Zhang T, Tong W, Liu H, Wang H, Li H (2021). Effect of welding thermal treatment on the microstructure and mechanical properties of nickel-based superalloy fabricated by selective laser melting. Mater. Sci. Eng. A..

[81] McEvily A (1977). Current aspects of fatigue. Met. Sci..

[82] Pippan R, Flechsig K, Riemelmoser FO (2000). Fatigue crack propagation behavior in the vicinity of an interface between materials with different yield stresses. Mater. Sci. Eng. A..

[83] Sun D, Huo J, Chen H, Dong Z, Ren R (2022). Experimental study of fretting fatigue in dovetail assembly considering temperature effect based on damage mechanics method. Eng. Fail. Anal..

[84] Wang Z, Qiang H (2022). Mechanical properties of thermal aged HTPB composite solid propellant under confining pressure. Def. Technol..

[85] Gao T, Li C, Wang Y, Liu X, An Q, Li HN, Zhang Y, Cao H, Liu B, Wang D, Said Z, Debnath S, Jamil M, Ali HM, Sharma S (2022). Carbon fiber reinforced polymer in drilling: From damage mechanisms to suppression. Compos. Struct..

